# Conformation and Aromaticity Switching in a Curved Non‐Alternant sp^2^ Carbon Scaffold

**DOI:** 10.1002/anie.202010077

**Published:** 2020-09-24

**Authors:** Chongwei Zhu, Kazutaka Shoyama, Frank Würthner

**Affiliations:** ^1^ Institut für Organische Chemie and Center for Nanosystems Chemistry (CNC) Universität Würzburg Am Hubland 97074 Würzburg Germany

**Keywords:** aromaticity, annulation, azulene, intermolecular mixed-valence, polycyclic aromatic hydrocarbons

## Abstract

A curved sp^2^ carbon scaffold containing fused pentagon and heptagon units (**1**) was synthesized by Pd‐catalyzed [5+2] annulation from a 3,9‐diboraperylene precursor and shows two reversible oxidation processes at low redox potential, accompanied by a butterfly‐like motion. Stepwise oxidation produced radical cation **1** ^.+^ and dication **1** ^2+^. In the crystal structure, **1** exhibits a chiral *cisoid* conformation and partial π‐overlap between the enantiomers. For the radical cation **1** ^.+^, a less curved *cisoid* conformation is observed with a π‐dimer‐type arrangement. **1** ^2+^ adopts a more planar structure with *transoid* conformation and slip‐stacked π‐overlap with closest neighbors. We also observed an intermolecular mixed‐valence complex of **1**⋅(**1** ^.+^)_3_ that has a huge trigonal unit cell [(**1**)_72_(SbF_6_)_54_⋅(hexane)_101_] and hexagonal columnar stacks. In addition to the conformational change, the aromaticity of **1** changes from localized to delocalized, as demonstrated by AICD and NICS(1)_*zz*_ calculations.

## Graphical abstract

A curved non‐alternant polycyclic aromatic hydrocarbon (PAH) containing fused heptagon and pentagon units is presented, as well as its conformational changes upon stepwise oxidation. Single‐crystal X‐ray analyses for neutral, cationic, dicationic, and a mixed valence species **1**⋅(**1** ^.+^)_3_ enabled the observed functional properties to be related to the molecular and supramolecular structures.

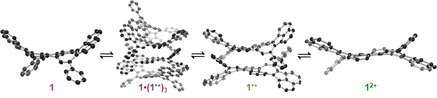

Curved polycyclic aromatic hydrocarbons (PAHs) have attracted considerable interest over the last decade in the context of research on carbon‐based materials.^[1]^ In contrast to their flat counterparts,^[2]^ curved π‐conjugated PAHs exhibit better solubility, a wealth of structural topologies including chirality,^[3]^ and may enable pronounced dynamic structural changes upon photoexcitation or electrochemical reduction/oxidation.^[4]^ With this motivation, incorporation of non‐hexagonal rings into polybenzenoid π‐networks has evolved as an important strategy toward curved PAHs with different curvatures. An embedded four‐ or five‐membered ring in a hexagonal π‐network induces a positive curvature, while inclusion of a heptagon or octagon produces a negative curvature.^[3a]^ PAHs that contain individual pentagon and heptagon in a hexagonal network of sp^2^ carbons may have both positive and negative curvatures,^[5]^ while those that incorporate fused pentagon and heptagon afford planar or contorted π‐scaffolds as demonstrated in recently reported azulene‐embedded polyaromatics.^[6]^ Compared to widely studied positively curved PAHs,^[7]^ saddle‐shaped ones with negative curvature were less developed until recently due to synthetic challenges.[^8^, ^9^]

Cycloheptatrienyl (C_7_H_7_) is a non‐aromatic seven‐membered ring with a nonplanar structure. Upon loss of an electron, it produces a non‐benzenoid tropylium cation (C_7_H_7_^+^). This intriguing π‐system has a planar geometry with 6 π‐electrons fully delocalized over the whole ring, which satisfies Hückel's [4*n*+2] rule for aromaticity. We reason that the negatively curved PAHs bearing heptagons comply with the identical rule upon oxidations, which would cause reorganization of their geometries and pronounced redistribution of the π‐electrons. These features may play decisive roles not only for their reactivity^[10]^ but also performance in battery applications^[11]^ or as organic semiconductors.^[12]^ Conformational switching can also lead to distinct stacking modes in the solid‐state that strongly affect the electronic properties of materials.^[13]^ However, to the best of our knowledge, a fundamental study concerning the structural and electronic properties of negatively curved π‐structures at different oxidation states remains unexplored.

Herein, we report a new negatively curved non‐alternant sp^2^ carbon scaffold, and its structural changes upon oxidation to the radical cation and dication species by stepwise oxidation. The electronic properties, magnetic aromaticity, conformational variations, and stacking arrangements in the solid‐state of this saddle‐shaped PAH at different oxidation states are investigated both experimentally and theoretically.

In our previous work,^[14]^ we developed a synthetic strategy for heptagon‐containing PAHs that formally include two pleiadiene moieties.^[15]^ In order to achieve a more electron‐rich curved PAH for studying oxidation states, PAH **1** that contains fused pentagon and heptagon was thus synthesized by the above‐mentioned method.^[14]^ Thus, a two‐fold palladium‐catalyzed [5+2] annulation of 3,9‐diboraperylene^[16]^ and 1,2‐dibromoacenaphthylene afforded azulene‐embedded PAH **1** in an isolated yield of 15 % (Scheme 1). PAH **1** exhibits high solubility in common organic solvents and good stability in the solid‐state with no significant decomposition after several weeks under ambient conditions.

**Scheme 1:**
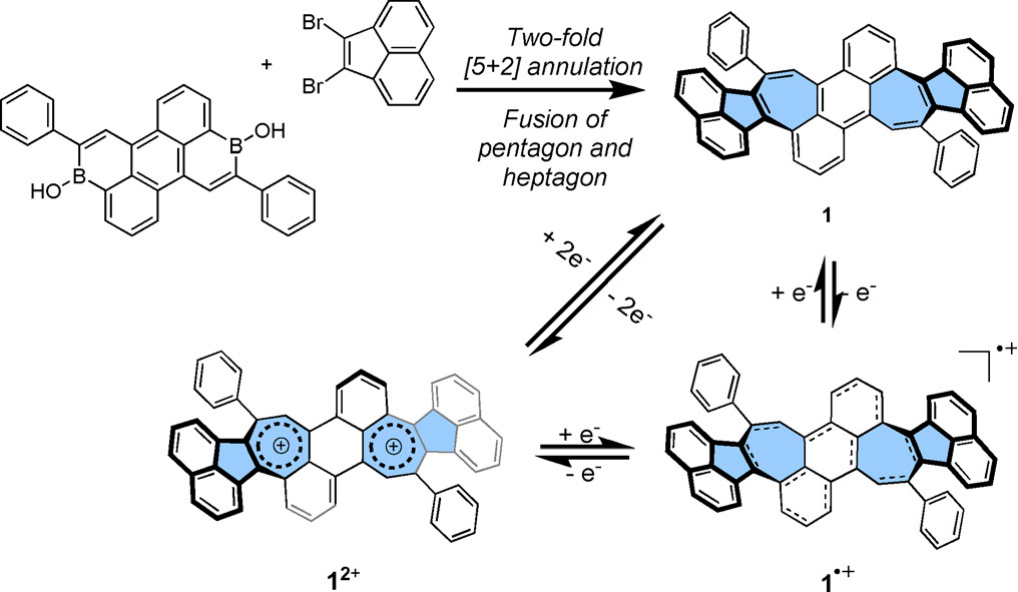
Synthesis of **1** by two‐fold Pd‐catalyzed [5+2] annulation and formation of its radical cation **1** ^.+^ and dication **1** ^2+^ by stepwise oxidation. Reaction conditions: [Pd_2_(dba)_3_]⋅CHCl_3_ (6 mol %), ^*t*^Bu_3_PHBF_4_ (14 mol %), Cs_2_CO_3_ (6.6 equiv), H_2_O (20 equiv), ^*t*^AmOH, 100 °C, N_2_, 48 h. Yield of isolated product: 15 %. Chemical oxidation conditions: NO⋅SbF_6_, dichloromethane, N_2_, r.t., 5 min.

We first investigated the redox properties of curved PAH **1** by cyclic voltammetry and square wave voltammetry (Figure 1). The cyclic voltammogram of **1** shows two low‐lying reversible oxidation processes at 0.00 V and 0.20 V (vs. Fc^+/0^). This eagerness toward oxidation arises from the two embedded heptagons (Figure S8, Table S12). Moreover, a *quasi*‐reversible reduction process at −1.78 V and an irreversible one at −1.92 V were observed. Motivated by the well‐defined reversibility of the oxidation processes of **1**, we next turned our attention to the stepwise chemical oxidation of **1** with NO⋅SbF_6_ (Scheme 1). Single‐electron chemical oxidation with one equivalent of NO⋅SbF_6_ afforded radical cation **1** ^.+^. Addition of another equivalent of NO⋅SbF_6_ to the solution of **1** ^.+^ produced dication **1** ^2+^ that can also be obtained directly by two‐electron oxidation of **1**. It's noteworthy that both of the two cationic species can convert back to the neutral species **1** upon reduction with triethylamine. The structures of charged species **1** ^.+^ and **1** ^2+^ as well as neutral species **1** could be unequivocally confirmed by NMR spectroscopy (for **1** and **1** ^2+^) and single crystal X‐ray crystallography. Likewise, the optical properties of all isolated species of **1** could be examined by UV/Vis absorption spectroscopy (Figure S5).

**Figure 1:**
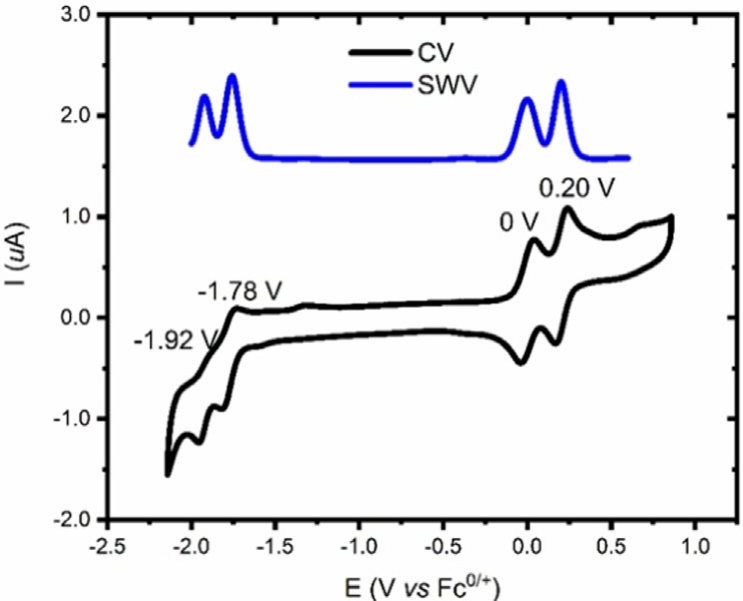
Cyclic and square‐wave voltammetry traces of **1**. Electrochemical measurements were carried out at 4.4×10^−4^ M of **1**, 0.1 M of *n*‐Bu_4_NPF_6_ in degassed CH_2_Cl_2_ at 298 K with a scan rate of 100 mV s^−1^. Half‐wave potentials were calculated from CV and SWV with ferrocene/ferrocenium (Fc/Fc^+^) as an internal reference.

Crystallographic analyses of **1**, **1** ^.+^, and **1** ^2+^ revealed their molecular conformations and intermolecular interactions in the solid‐state (Figure 2).^[17]^ The crystal structure of the neutral species **1** shows a twisted saddle‐shaped *cisoid* conformation (Figure 2 b), that results from the distorted geometry of the seven‐membered ring and steric hindrance between the anthracene and naphthalene moieties. The dihedral angle between the mean planes of two acenaphthalene moieties is 86.3°. Similarly, radical cation **1** ^.+^ exhibits a *cisoid* conformation with a larger dihedral angle of 110.5° or 90.3° (Figure 2 e). The counter anion (SbF_6_^−^) stays beside the central anthracene core and has no close contact with the seven‐membered rings (Figure 2 d, e). In contrast, dication **1** ^2+^ adopts a *trans*oid conformation in the crystal (Figure 2 h) with a dihedral angle of 151.6° between the mean planes of the acenaphthalene and anthracene units. Two counter anions (SbF_6_^−^) reside closely to the heptagons and are slightly shifted towards the central benzene ring, suggesting the presence of tropylium‐like cations. This observation is also in line with the DFT calculated charge distribution and electrostatic potential maps where the positive charges are mainly located on the heptagons (Figure S8, Table S12). The *cis*‐ and *trans*‐conformations of **1**, **1** ^.+^ and **1** ^2+^ were also studied by DFT calculations at B3LYP/6‐31+G(d) level of theory. The *C*_2_‐symmetric *cisoids* of **1**, **1** ^.+^ and **1** ^2+^ are lower in energy than the corresponding *C_i_*‐symmetric *transoids* by 12.1, 7.2, and 10.3 kJ mol^−1^, respectively. This suggests that the *cis*‐conformations are more energetically favorable in vacuum, and the *trans*‐conformation of **1** ^2+^ in the crystal state might arise from the effects of the two counterions at the opposite sides of π‐scaffold and the crystal packing forces.^[18]^

**Figure 2:**
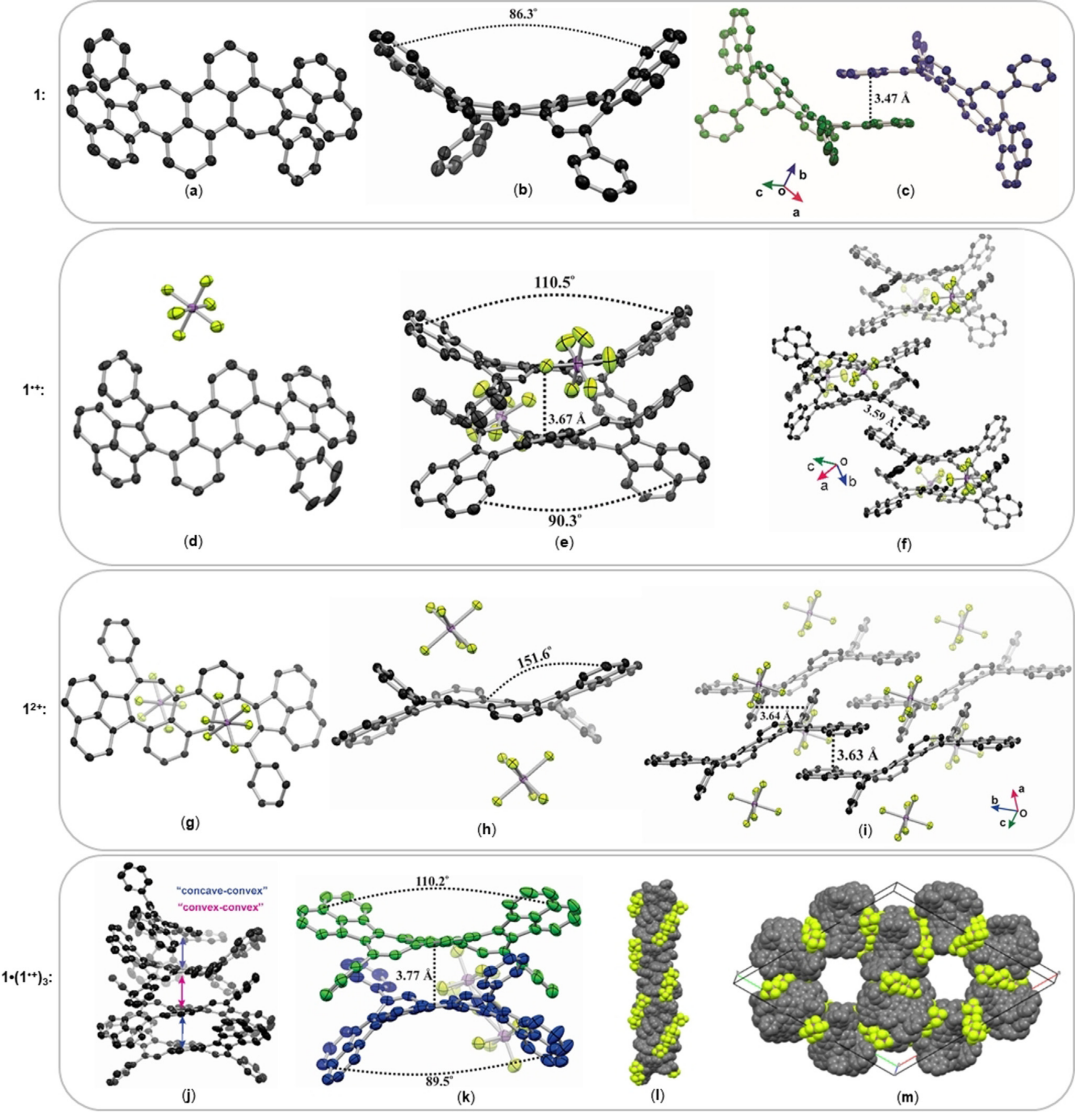
a–i) X‐ray crystal structures of neutral species **1** (a–c), radical cation **1** ^.+^ (d–f), dication **1** ^2+^ (g–i), shown as the front view (left), side view (middle), and crystal packing (right). j–m) X‐ray crystal structures of mixed valence species **1**⋅(**1** ^.+^)_3_, showing the selected repeating unit (j); side view (k); columnar π‐stacks (l); and crystal unit cell from top view (m). Thermal ellipsoids shown at 50 % probability; solvent molecules and hydrogen atoms are omitted for clarity. C dark grey, F yellow, Sb magenta.

Interestingly, upon partial oxidation of **1** with NO⋅SbF_6_, we obtained crystals of a mixed‐valence (MV) species **1**⋅(**1** ^.+^)_3_ that consists of 1/4 equivalent of neutral species **1** and 3/4 equivalents of radical cation **1** ^.+^ (Figure 2 j–m). This MV complex **1**⋅(**1** ^.+^)_3_ has similar dihedral angles as the radical cation **1** ^.+^ (Figure 2 k). The crystal structure of **1**⋅(**1** ^.+^)_3_ features a huge trigonal unit cell (91 777(9) Å^3^) that contains 72 molecules with *cisoid* conformation, 54 SbF_6_^−^ counter anions and 101 disordered hexane molecules, [(**1**)_72_(SbF_6_)_54_⋅(hexane)_101_]. Remarkably, we could also identify the MV species in solution by UV/Vis/NIR absorption spectroscopy which suggests the formation of supramolecular adducts of this composition already in solution (Figure S4).

The packing arrangements of the series of crystals **1**, **1**⋅(**1** ^.**+**^)_3_, **1** ^.+^, **1** ^2+^ reflect their oxidation state (Figure 2 c, f, i, m). In the crystal packing, the neutral species **1** has conformational chirality and exists as a pair of enantiomers (Figure 2 c, highlighted in green and blue). These two enantiomers have partial slip‐stacked π‐π overlap between two acenaphthylene moieties with a distance of 3.47 Å. Similarly, dication **1** ^2+^ shows such “head‐to‐tail” π‐π interaction through two acenaphthylene units separated by 3.63 Å (Figure 2 i). Unlike **1** and **1** ^2+^, radical cation **1** ^.+^ shows a cofacial π‐dimer arrangement that might support the stability of the radical species (Figure 2 e, f).^[19]^ The centroid‐centroid distance between two central benzene rings was measured as 3.67 Å. These π‐dimers pack into staggered arrangements through partial π‐π interactions of adjacent acenaphthylene units with an interplanar distance of 3.59 Å. The most intriguing structure formed by the mixed‐valence species **1**⋅(**1** ^.+^)_3_ reveals a self‐assembly into an infinite columnar arrangement through “concave‐convex” and “convex‐convex” π‐π‐stacking modes with an average distance of 3.77 Å for two contiguous π‐systems in each column.[^7c^, ^20^] Furthermore, these columns self‐organize into hexagonal arrays, in which the void space of the hollow hexagons is occupied by disordered solvent molecules, and the counterions SbF_6_^−^ are evenly intercalated between the columns (Figure 2 m, S6).

To explore the aromaticity and electronic structures of **1** at different oxidation states, we performed anisotropy of the induced current‐density (AICD)^[21]^ and nucleus‐independent chemical shift (NICS) calculations^[22]^ at (U)B3LYP/6–31+G(d) level of theory (Figure 3). The AICD plot of neutral species **1** shows diamagnetic ring currents on the two terminal naphthalene and central anthracene moieties with large negative NICS(1)_*zz*_ values, suggesting a localized aromaticity of the respective moiety. The heptagons of neutral **1** have counter‐clockwise paramagnetic ring currents and a large positive NICS(1)_*zz*_ value of 17.67, indicating their strong antiaromaticity.^[6c]^ The antiaromaticity of heptagons is evident, since the shielded protons on the seven‐membered rings resonate at 7.17 ppm in ^1^H NMR spectrum (Figure S1). Moreover, the small positive NICS(1)_*zz*_ value (6.89) for pentagons suggests weak antiaromaticity, different from that in pristine acenaphthylene in which the pentagon has a non‐aromatic character.^[23]^ In contrast to **1**, the dication **1** ^2+^ exhibits a clockwise diamagnetic ring current alongside the periphery of the half π‐conjugated framework (Figure 2 c). In accordance to this, the central benzene ring becomes antiaromatic with a positive NICS(1)_*zz*_ value of 11.25. The heptagons and pentagons of **1** ^2+^ have weak aromaticity with NICS(1)_*zz*_ values of −4.0 and −4.5, respectively. This result can be further supported by the ^1^H NMR spectrum (Figure S1), in which the chemical shift of the proton on heptagonal rings is deshielded from 7.17 ppm in **1** to 9.04 ppm in **1** ^2+^.^[24]^ Radical cation **1** ^.+^ shows fragmental delocalized aromaticity with clockwise ring currents over naphthalene and the marginal benzene rings of the anthracene moiety. This is because of a very weak (anti)aromaticity of the pentagon, heptagon and central benzene rings of **1** ^.+^. These variations of aromaticity indicate that this curved π‐scaffold undergoes delocalization of π‐electrons along with conformational changes upon stepwise oxidation.

**Figure 3:**
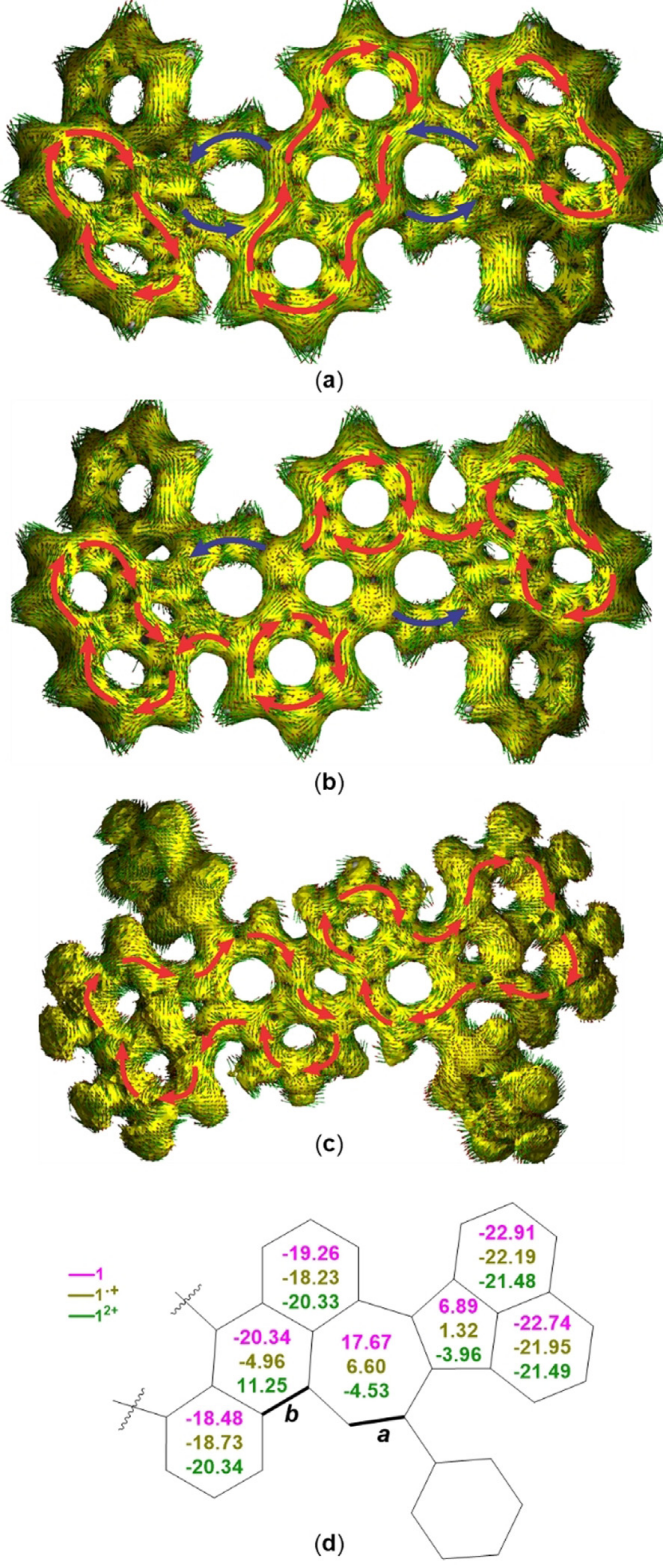
a–c) Calculated AICD plots (isovalue: 0.05) of neutral species **1** (a); radical cation **1** ^.+^ (b); dication **1** ^2+^ (c). Arrows in red represent clockwise ring current, arrows in blue represent counter clockwise ring current. d) NICS(1)_*zz*_ values of **1** (pink), **1^.+^** (yellow green) and **1** ^2+^ (dark green) at (U)B3LYP/6‐31+G(d) level of theory; ***a*** and ***b*** are selected bonds for bond‐length analysis.

The calculated changes in aromaticity are supported by the bond length analyses of the molecular structures of **1** in the crystals at different oxidation states (Figure S7, Tables S6,7). Thus, the length of bond ***a*** changes from 1.353(3) Å for **1** to 1.388(9) Å for **1** ^2+^. This corresponds to a shift from a conjugated double bond (‐C=C‐, 1.345 Å) to an aromatic bond (‐C_Ar_≃C_Ar_‐, 1.384 Å).^[25]^ Concomitantly, the bond length of ***b*** becomes longer from 1.432(3) Å for **1** to 1.478(9) Å for **1** ^2+^. This can be interpreted as a shift from an equivalent bond of anthracene (‐C9≃C9a‐, 1.400 Å) to a single bond between two aromatic carbons (‐C_Ar_–C_Ar_‐, 1.487 Å).^[25]^ All these bond length changes as well as other characteristic bond lengths of the seven‐membered and central benzene rings in **1** have systematic variations as the oxidation state increases. It's worth mentioning that we observed two crystallographic isomers of the MV species **1**⋅(**1** ^.+^)_3_ that derived from the intra‐crystal charge delocalization of neutral and radical cation species. On the basis of bond length analysis (Table S6), one (Figure 2, highlighted in blue, MV_I_) is closer to neutral species and the other (highlighted in green, MV_II_) is closer to radical cation (Figure 2 k).^[26]^

In summary, we introduced a negatively curved non‐alternant PAH containing two fused pentagon and heptagon units and elucidated its structural and functional changes upon stepwise chemical oxidation. X‐ray crystallographic analyses revealed how this saddle‐shaped PAH changes its conformation and aromatic ring currents as the oxidation state increases. Thus, along with the butterfly‐like conformational motions from *cisoid* to *transoid*, the magnetic aromaticity alters from localization to delocalization according to AICD plots and NICS(1)_*zz*_ calculations. Our study on this curved non‐alternant PAH serves to interrelate the effects of charges on the structural and electronic properties as well as packing preferences of nanographenes containing odd‐membered ring defects.

## Conflict of interest

The authors declare no conflict of interest.

## Supporting information

As a service to our authors and readers, this journal provides supporting information supplied by the authors. Such materials are peer reviewed and may be re‐organized for online delivery, but are not copy‐edited or typeset. Technical support issues arising from supporting information (other than missing files) should be addressed to the authors.

Supplementary
