# Supplementary material for: Conformation and Aromaticity Switching in a Curved Non‐Alternant sp2 Carbon Scaffold
Source: Angew Chem Int Ed Engl. 2020 Sep 24;59(48):21505–9. doi: 10.1002/anie.202010077 (PMC7756343; doi:10.1002/anie.202010077)
Supplement: Supplementary file 1 — Supplementary [file ANIE-59-21505-s001.pdf]

Angewandte Chemie
Eine Zeitschrift der Gesellschaft Deutscher Chemiker

Supporting Information

Conformation and Aromaticity Switching in a Curved Non-Alternant sp^2 Carbon Scaffold

*Chongwei Zhu, Kazutaka Shoyama, and Frank Würthner**

anie_202010077_sm_miscellaneous_information.pdf

Table of Contents

Materials and Methods.. ....S3

Experimental Procedures……………………………………………………………………………..…...............…....……..S4

Charge analysis of **1** and **1**$^{2+}$ based on NMR spectra……………………:…………………………………………….S6

UV-vis-NIR Absorption Spectra.. ....S7

X-ray Crystallographic Data.. ....S10

Bond Lengths Analysis.. ....S15

DFT-computational Studies and Theoretical Analyses.. ....S21

NMR Spectra.. ....S35

References.. ....S41

Materials and Methods

General remarks: Commercially available chemicals of tri-*t*-butylphosphonium tetrafluoroborate, *t*-amyl alcohol, Cs_2CO_3, nitrosonium hexafluoroantimonate, acenaphthylene were purchased and directly used without further purification. Column chromatography was performed with commercial glass columns using silica gel 60M (particle size 0.04-0.063 mm). All other reagents and solvents were obtained from commercial sources and used without further purification. All the reactions were carried out in dry Schlenk tubes under protection of nitrogen.

UV-Vis absorption spectroscopy was measured on Jasco V-670 and V-770 spectrophotometers.

NMR spectroscopy was measured on Bruker Avance III HD 400 and Bruker Avance III HD 600 spectrometers. Chemical shifts are given in ppm relative to $SiMe_4$, multiplicity (s = singlet, d = doublet, t = triplet, m = multiplet), coupling constants (*J*) are shown in Hertz (Hz). All the peaks were assigned according to ^1H, ^{13}C, ^1H-^1H COSY, ^1H-^1H NOESY, ^1H-^{13}C HSQC, and ^1H-^{13}C HMBC NMR spectra.

High resolution mass spectrometry was measured on a Bruker Daltonics microTOF focus instrument; MS was measured on a Bruker Daltonics ultrafleXtreme mass spectrometer.

Cyclic and square wave voltammetry was measured with a standard commercial electrochemical analyzer (EC epsilon; BAS Instruments, UK) with a three-electrode single-compartment cell. Tetrabutylammonium hexafluorophosphate (*n*-Bu_4NPF_6) was applied as supporting electrolyte, ferrocene (Fc) as an internal standard for the calibration of potentials, Ag/AgCl as reference electrode, Pt disc and Pt wire as working and auxiliary electrodes, respectively. Cyclic voltammetry (CV) and square wave voltammetry (SWV) were performed at a scan rate of 100 mV/s at room temperature. The redox potentials were referenced against the ferrocenium/ferrocene (Fc+/Fc) redox couple.

Single crystal X-ray analysis was carried out on Bruker D8 Quest Kappa diffractometers with a PhotonII CMOS detector and multi-layered mirror monochromated CuKα radiation.

Theoretical calculations: Geometry optimizations, $NICS(1)_{zz}$ values, excited state studies, and AICD analysis were performed using density functional theory (DFT) at the level of B3LYP/6-31+G(d) as implemented in the Gaussian 16 program. The AICD software package was provided by Prof. R. Herges. Charged species for DFT calculations were conducted by using only π-scaffolds without counterions.

Experimental Procedures

Experimental procedure for the synthesis of 1.

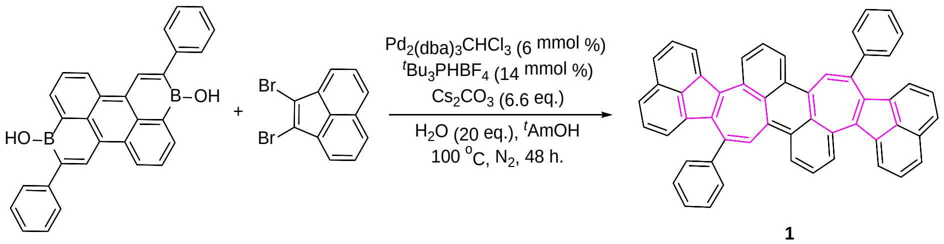

1

A solid mixture of 3,9-diboraperylene[1,2] (30 mg, 0.0690 mmol, 1 eq.), 1,2-dibromoacenaphthylene[3] (64 mg, 0.2100 mmol, 3.0 eq.), $Pd_2(dba)_3$•$CHCl_3$ (4.3 mg, 0.0041 mmol, 0.060 eq.), ^{t}BuP•HBF_4 (2.9 mg, 0.0097 mmol, 0.14 eq.), and Cs_2CO_3 (148 mg, 0.4600 mmol, 6.6 eq.) in an oven-dried Schlenk tube was evacuated and refilled back with N_2 three times, and then ^{t}AmOH (12 mL) and H_2O (25 µL, 1.4000 mmol, 20 eq.) were added with syringes. The reaction mixture was stirred at room temperature for 1 hour prior to heating up to 100 °C for 48 hours. After cooling down to room temperature, the reaction solution was diluted with EtOAc (50 mL) and filtered through celite®. The filtrate was concentrated to dryness under reduced pressure, and the residue was purified by column chromatography on silica gel eluting with mixture solvents of hexane/DCM (3:1 in *V/V*) and then hexane/EtOAc (6:1 in *V/V*), and by washing with hexane, affording the product **1** as a dark violet solid with a yield of 15% (7 mg). Single crystals of **1** suitable for crystallographic measurement were obtained by slow diffusion of hexane into $CHCl_3$ at 8 °C.

^{1}H and ^{13}C NMR data of 1:

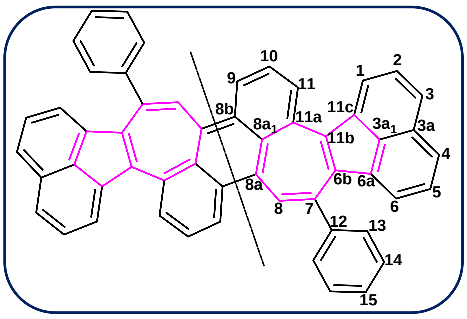

^{1}H NMR (600 MHz, CD_2Cl_2, 295 K) δ 8.27 – 8.26 (dd, *J* = 8.74, 0.85 Hz, 2H, *H9*), 7.92 – 7.91 (dd, *J* = 7.02, 0.9 Hz, 2H, *H11*), 7.89 (d, *J* = 7.09 Hz, 2H, *H1*), 7.85 (d, *J* = 8.0 Hz, 2H, *H3*), 7.77 – 7.55 (m, 6H, *H4*, *H13*), 7.69 – 7.66 (dd, *J* = 8.68, 7.02 Hz, 2H, *H10*), 7.53 (dd, *J* = 8.01, 7.20 Hz, 2H, *H2*), 7.51 – 7.47 (m, 6H, *H14*, *H15*), 7.31 (dd, *J* = 8.16, 7.16 Hz, 2H, *H5*), 7.18 (s, 2H, *H8*), 6.66 (d, *J* = 7.03 Hz, 2H, *H6*).

^{13}C{^{1}H} NMR (151 MHz, CD_2Cl_2, 295 K): 143.32 (*C11a*), 143.29 (*C12*), 140.44 (*C7*), 139.95 (*C11c*), 138.80 (*C6b*), 138.67 (*C8a*), 138.32 (*C6a*), 134.34 (*C8*), 133.34 (*C11b*), 132.15 (*C8a$_1$*), 130.12 (*C8b*), 129.89 (*C3a$_1$*), 129.23 (*C13*), 128.80 (*C3a*), 128.69 (*C14*), 128.50 (*C15*), 128.40 (*C3*), 127.81 (*C5*), 127.77 (*C2*), 127.63 (*C4*), 126.78 (*C11*), 126.66 (*C10*), 125.77 (*C6*), 123.55 (*C9*), 122.87 (*C1*).

MS (MALDI-TOF, positive mode): m/z: $[M]^+$ calculated for $C_{54}H_{30}$: 678.2342, found: 678.2348.

ESI-HRMS (positive mode): m/z: $[M]^+$ calculated for $C_{54}H_{30}^+$: 678.2342, found: 678.2361.

UV/Vis (CH_2Cl_2, 295 K): λ = 559 nm (ε_{559nm} = 10500 L•mol^{-1}•cm^{-1}), 355 nm (ε_{355nm} = 15000 L•mol^{-1}•cm^{-1}), 298 nm (ε_{298nm} = 25000 L•mol^{-1}•cm^{-1}).

Cyclic voltammetry (2.5 × 10^{-4} M, 0.1 M *n*-Bu_4NPF_6 in CH_2Cl_2 at 295 K, $E^{½}_{Redox}$ *vs*. Fc^+/Fc): $E^{½}_{Ox1}$ = 0.00 V, $E^{½}_{Ox2}$ = 0.20 V, $E^{½}_{Red1}$ = −1.78 V, $E^{½}_{Red1}$ = −1.92 V.

Preparation of radical cation $1^{•+}$ and dication 1^{2+} by chemical oxidation.

1) Preparation of radical cation **1**$^{•+}$

To a solution of **1** (2.6 mg, 0.0038 mmol, 1.0 eq.) in dry CH_2Cl_2 (2 mL) in an oven-dried Schlenk tube was added the solution of NO•SbF_6 (1 mg, 0.0038 mmol, 1.0 eq.) in dry acetonitrile (50 µL) at room temperature under nitrogen. The reaction was stirred for 5 min along

with a color change of the solution from dark violet to yellow green. The reaction mixture was concentrated under reduced pressure to afford radical species **1**$^{•+}$ as a brown solid. The product **1**$^{•+}$ was directly used without further purification. Single crystals of **1**$^{•+}$ were grown by slow evaporation/diffusion in CH_2Cl_2/hexane at 8 °C in the nitrogen-filled glovebox.

2) Preparation of dication **1**$^{2+}$

To a solution of **1**$^{•+}$ (0.0038 mmol, 1.0 eq.) in dry CH_2Cl_2 (2 mL) in an oven-dried Schlenk tube was further added NO•SbF_6 (1 mg, 0.0038 mmol, 1.0 eq.) in dry acetonitrile (50 μL) at room temperature under nitrogen. After the reaction mixture was stirred for 5 min, along with color change of the solution from yellow green to dark green. The reaction mixture was concentrated under reduced pressure to give dication **1**$^{2+}$ as a dark green solid. The product **1**$^{2+}$ was directly used without further purification. Alternatively, the dication **1**$^{2+}$ can also be synthesized by direct addition of two equivalents of NO•SbF_6 to a solution of **1** in CH_2Cl_2. Single crystals of **1**$^{2+}$ were obtained in CH_2Cl_2 at −20 °C under N_2 protection.

Radical cation **1**$^{•+}$:

UV/Vis/NIR (CH_2Cl_2, 295 K): λ = 1374 nm (ε_{1374nm} = 4100 L•mol^{-1}•cm^{-1}), 937 nm (ε_{937nm} = 30000 L•mol^{-1}•cm^{-1}), 377 nm (ε_{377nm} = 39000 L•mol^{-1}•cm^{-1}).

A mass spectrum of radical cation **1**$^{•+}$ could not be recorded due to its instability under ambient conditions.

^{1}H and ^{13}C NMR data of 1^{2+}:

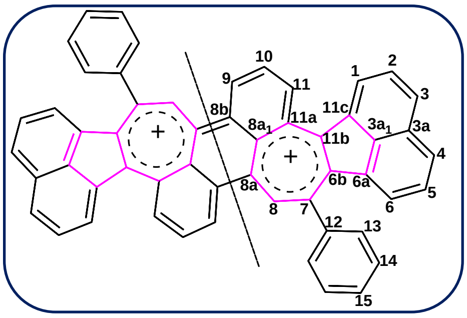

^{1}H NMR (400 MHz, CD_2Cl_2, 295 K) δ 9.42 – 9.44 (dd, *J* = 8.38, 1.00 Hz, 2H, *H11*), 9.07 (d, *J* = 7.65 Hz, 2H, *H9*), 9.03 (s, 2H, *H8*), 8.92 (d, *J* = 7.39 Hz, 2H, *H1*), 8.75 (t, *J* = 8.38 Hz, 2H, *H10*), 8.68 (d, *J* = 7.95 Hz, 2H, *H3*), 8.41 (d, *J* = 7.74 Hz, 2H, *H4*), 8.24 (t, *J* = 7.74 Hz, 2H, *H2*), 8.08 – 8.04 (m, 4H, *H13*), 7.88 (m, 2H, *H15*), 7.82 (m, 4H, *H14*), 7.77 (t, *J* = 7.80 Hz, 2H, *H5*), 7.26 (d, *J* = 6.96 Hz, 2H, *H6*).

^{13}C{^{1}H} NMR (101 MHz, CD_2Cl_2, 295 K): 165.42 (*C7*), 162.92 (*C11a*), 150.85 (*C8a*), 148.37 (*C6b*), 142.24 (*C8*), 140.82 (*C11*), 139.94 (*C12*), 138.76 (*C9*), 138.48 (*C3*), 136.80 (*C8b*), 136.35 (*C11c*), 136.23 (*C1*), 135.98 (*C10*), 135.18 (*C11b*), 134.31 (*C8a$_1$*), 133.36 (*C15*), 133.30 (*C4*), 133.06 (*C6a*), 132.69 (*C3a$_1$*), 131.57 (*C6*), 130.91 (*C2*), 130.50 (*C13*), 130.13 (*C5*), 129.98 (*C3a*), 129.86 (*C14*).

UV/Vis (CH_2Cl_2, 295 K): λ = 681 nm (ε_{681nm} = 26000 L•mol^{-1}•cm^{-1}), 461 nm (ε_{461nm} = 31000 L•mol^{-1}•cm^{-1}).

A mass spectrum of dication **1**$^{2+}$ could not be recorded due to its instability under ambient conditions.

Charge analysis of 1 and 1^{2+} based on NMR spectra

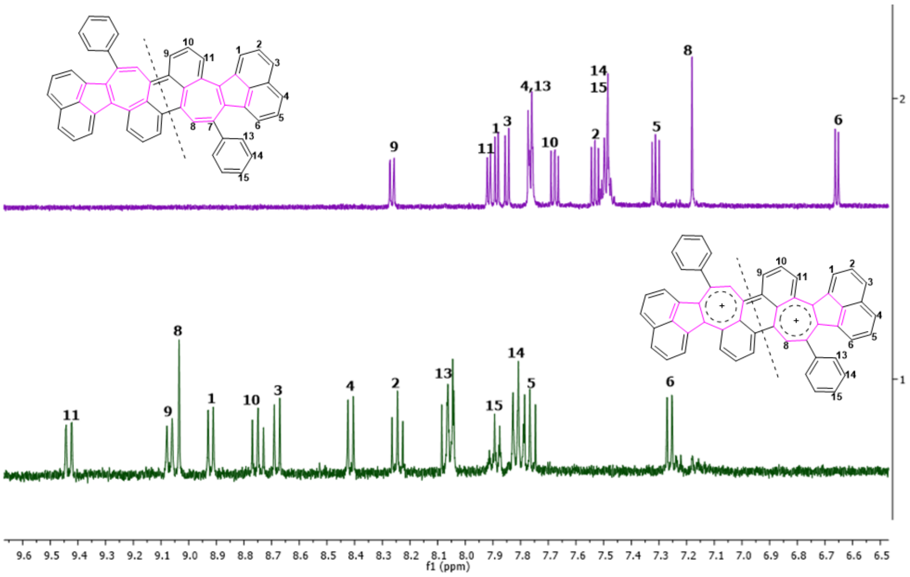

Figure S1. The expanded aromatic region of the ^{1}H NMR spectra (400 MHz, CD_2Cl_2, 295 K) of neutral species **1** and dication **1**$^{2+}$ for comparison.

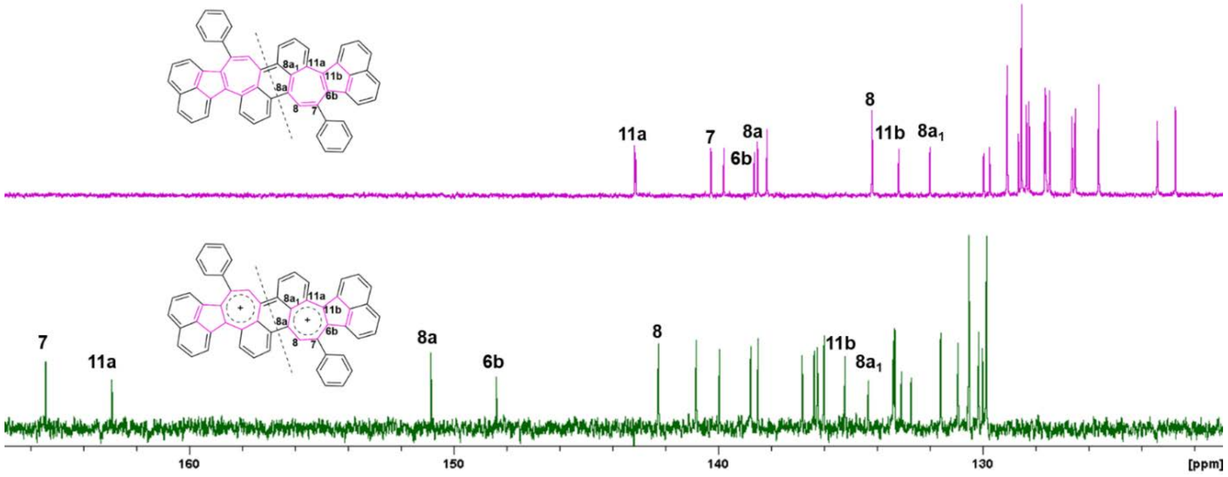

Figure S2. The expanded aromatic region of the ^{13}C NMR spectra (101 MHz, CD_2Cl_2, 295 K) of neutral species **1** and dication **1**$^{2+}$ for comparison.

Compared to the neutral counterpart **1**, all the peaks in ^{1}H NMR spectrum of dication **1**$^{2+}$ are highly deshielded. Particularly the proton (*H8*) of the heptagons significantly shifted from 7.18 ppm to 9.03 ppm. Similar variations were also observed in ^{13}C NMR spectra, in which the chemical shifts of carbon atoms of heptagonal rings are highly deshielded to downfield. These observations suggest gain in aromaticity due to the formation of tropylium-like cations.

Table S1. Local π-charge densities estimated by the chemical shift of ^{13}C NMR spectra[4–6] and calculated Mulliken partial atomic charges of neutral species **1** and dication **1**$^{2+}$.

Carbon atom	δ_C (**1**)^a	δ_C (**1**$^{2+}$)^a	$\Delta\delta_C{}^b$	Δq^c	q_M (**1**)^d	q_M (**1**$^{2+}$)^d	$\Delta q_M{}^e$
**1**	122.87	136.23	13.36	0.082	-0.311	-0.282	0.029
**2**	127.77	130.91	3.14	0.019	-0.582	-0.533	0.049
**3**	128.40	138.48	10.08	0.062	-0.241	-0.206	0.035
**3a**	128.80	129.98	1.18	0.007	0.359	0.354	-0.005
**3a$_1$**	129.89	132.69	2.80	0.017	-0.121	-0.094	0.027
**4**	127.63	133.30	5.67	0.035	-0.233	-0.042	0.191
**5**	127.81	130.13	2.32	0.014	-0.655	-0.650	0.005
**6**	125.77	131.57	5.80	0.036	-0.071	-0.151	-0.080
**6a**	138.32	133.06	-5.26	-0.032	0.296	0.292	-0.004
**6b**	138.80	148.37	9.57	0.059	-0.028	0.151	0.179
**7**	140.44	165.42	24.98	0.153	-0.478	0.291	0.769
**8**	134.34	142.24	7.90	0.048	-1.171	-0.751	0.420
**8a**	138.67	150.85	12.18	0.075	0.930	0.382	-0.548
**8a$_1$**	132.15	134.31	2.16	0.013	0.544	0.100	-0.444
**8b**	130.12	136.80	6.68	0.041	0.396	0.262	-0.134
**9**	123.55	138.76	15.21	0.093	-0.207	-0.212	-0.005
**10**	126.66	135.98	9.32	0.057	-0.784	-1.221	-0.437
**11**	126.78	140.82	14.04	0.086	-0.707	-0.585	0.122
**11a**	143.29	162.92	19.63	0.120	0.953	1.031	0.078
**11b**	133.34	135.18	1.84	0.011	-0.014	0.059	0.073
**11c**	139.95	136.35	-3.60	-0.022	0.440	0.403	-0.037
**12**	143.29	139.94	-3.35	-0.021	0.252	0.336	0.084
**13**	129.23	130.50	1.27	0.008	-0.405	-0.377	0.028
**14**	128.69	129.86	1.17	0.007	-0.181	-0.141	0.040
**15**	128.50	133.36	4.86	0.030	-0.231	-0.195	0.036

^{a} The chemical shifts (δ_C) of carbons are given in ppm. ^{b} $\Delta\delta_C = \delta_C$ (**1**$^{2+}$) − δ_C (**1**). ^{c} Local π-charge densities calculated by $\Delta\delta_C$ / K_c, where K_c is the average proportionality constant for carbon atoms. The sum of chemical shift changes of carbons from **1** to **1**$^{2+}$: $\sum\Delta\delta_C$ = 162.9 ppm. Accordingly, K_c is 162.9 ppm/electron, which is close to the proposed value of 160 ppm/electron.[4] ^{d} Partial atomic charge (Mulliken charge: q_M) calculated by DFT at B3LYP/6-31+G(d) level of theory. Molecular geometry was optimised by the same level of theory. ^{e} $\Delta q_M = q_M$ (**1**$^{2+}$) – q_M (**1**). Carbon atoms of heptagons are hightlighted in pink.

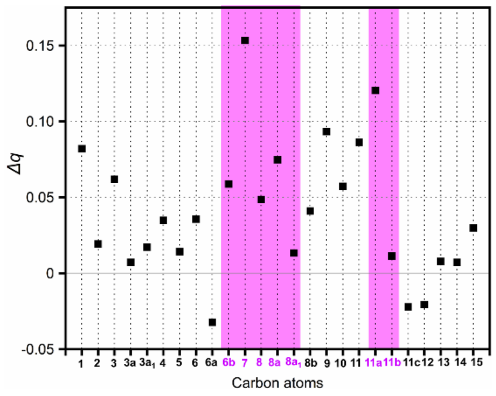

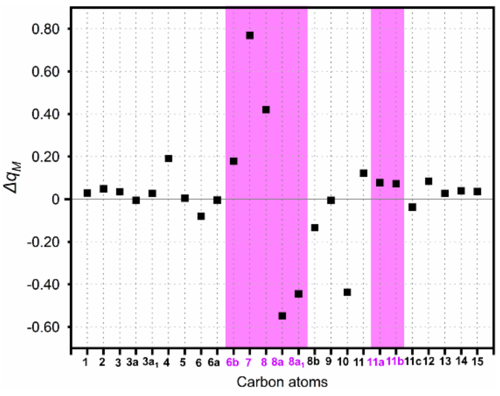

Figure S3. Plotted charge variations of carbon atoms from **1** to **1**$^{2+}$ based on the changes of ^{13}C NMR chemical shifts (left) and DFT-calculated Mulliken charges (right). Carbon atoms of heptagons are highlighted in pink.

Table S1 shows the charge changes of the carbon atoms of **1** upon loss of two π-electrons by chemical oxidation to dication **1**$^{2+}$. Figure S3 compares the experimental (Local π-charge density obtained from ^{13}C NMR chemical shifts) and theoretical (DFT-calculated Mulliken charges) values. In both cases similar trends of charge variations were found and larger charge changes were observed for the carbon atoms of heptagonal rings. These observations support the formation of tropylium-like cations.

UV/Vis/NIR Absorption Spectroscopy for the Chemical Titration with $NOSbF_6$

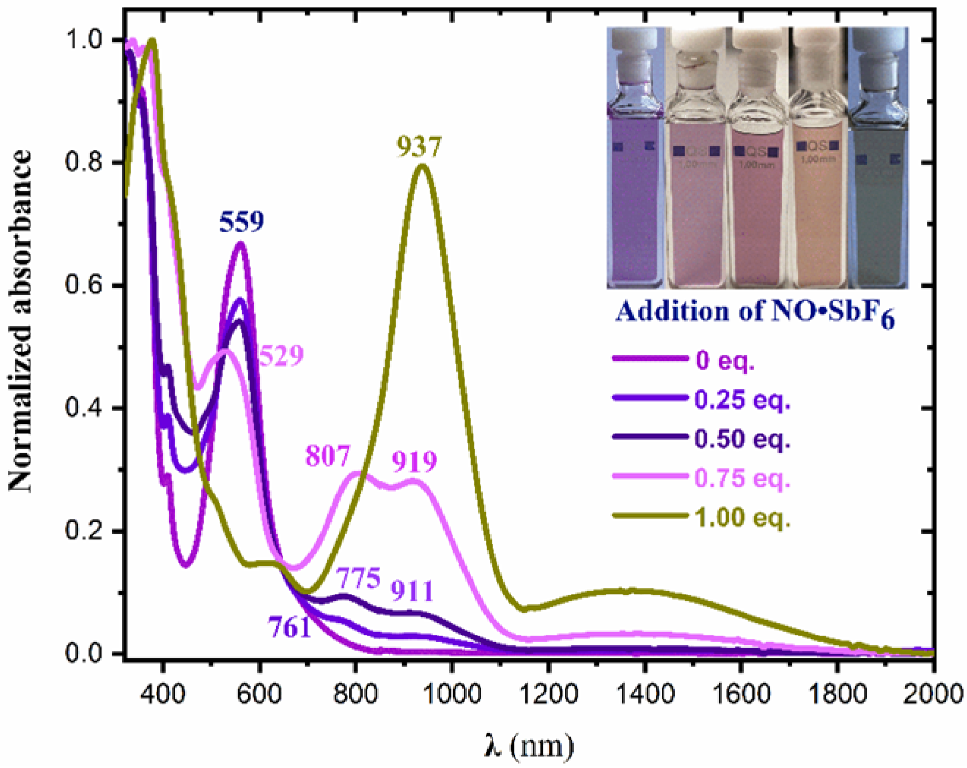

Figure S4. UV/Vis/NIR absorption spectra of **1** upon oxidation with different equivalents of NO•SbF_6. The absorption experiments were carried out in degassed CH_2Cl_2 under N_2 at 295 K.

Upon titration with NO•SbF_6 (less than 1 equiv.), the new absorption bands were observed in the range of 760–920 nm, which lie between the absorption band at 559 nm of neutral species **1** and that at 937 nm of radical cation **1**$^{\bullet+}$, suggesting the formation of complex between neutral species **1** and radical cation **1**$^{\bullet+}$. Further confirmation of this complex was obtained by X-ray crystallographic analysis.

UV/Vis/NIR absorption spectra of neutral species 1, radical cation $1^{•+}$ and dication 1^{2+}

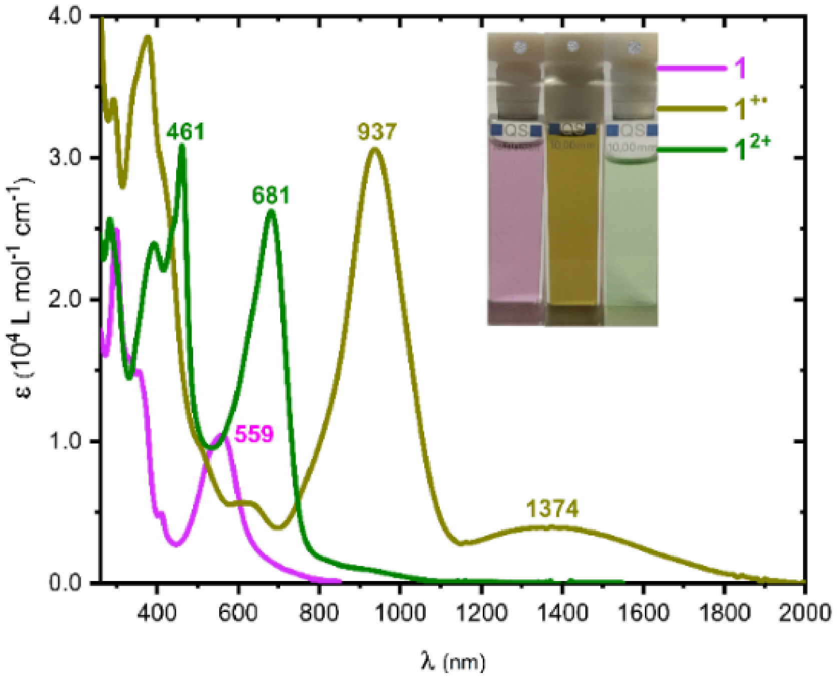

Figure S5. UV/Vis/NIR absorption spectra of **1**, **1**$^{•+}$ and **1**$^{2+}$ carried out in degassed CH_2Cl_2 at ambient temperature (1.11 × 10^{-5} M for **1**, violet line; 3.10 × 10^{-5} M for **1**$^{•+}$, yellow line; 9.67 × 10^{-6} M for **1**$^{2+}$, dark green line).

The optical properties of **1** and its charged species produced by chemical oxidation were examined by UV/Vis absorption spectroscopy (Figure S5) and TD-DFT calculations. In the UV/Vis spectrum, the neutral species **1** shows an absorption maximum at 559 nm (ε_{559nm} = 10 500 M^{-1}•cm^{-1}), along with a red tail that extends to 800 nm. According to our TD-DFT calculations we assign the main contribution of the absorption peak at 559 nm to the HOMO→LUMO+2 transition (S0→S3, f = 0.455) and the weak absorption at longer wavelengths to a partially allowed HOMO→LUMO transition (S0→S1, f = 0.060). The radical cation **1**$^{•+}$ exhibits two absorption bands in the near-infrared (NIR) spectral range with maxima at 1374 and 937 nm. The former absorption band is assigned to the α-HOMO→α-LUMO transition (S0→S1, f = 0.068), the latter mainly to a β-HOMO→β-LUMO transition (S0→S3, f = 0.434). Dication **1**$^{2+}$ displays a bathochromic shift and a significant increase in oscillator strength compared to the neutral species **1**. Thus, it has the main absorption maximum at 681 nm followed by a long tail extending into the NIR range. TD-DFT analyses assign this weak absorption to a combination of HOMO→LUMO and HOMO–2→LUMO transitions (S0→S1, f = 0.052).

X-ray Crystallographic Data

Table S2. Crystal data and structure refinement for the neutral species **1**.

CCDC Number	2018068	
Empirical formula	$C_{60}H_{44}$	
Moiety formula	$C_{54}H_{30}$, C_6H_{14}	
Formula weight	764.95	
Temperature	100(2) K	
Wavelength	1.54178 Å	
Crystal system	Monoclinic	
Space group	$P2_1/c$	
Unit cell dimensions	a = 21.8595(7) Å	α = 90°
	b = 12.3396(4) Å	β = 110°
	c = 16.1215(5) Å	γ = 90°
Volume	4084.5(2) $Å^3$	
Z	4	
Calculated density	1.244 Mg/m^3	
Absorption coefficient	0.531 mm^{-1}	
F(000)	1616	
Crystal size	0.30 × 0.18 × 0.02 mm^3	
Theta range for data collection	4.180 to 72.249°	
Limiting indices	$-26 \le h \le 26$, $-12 \le k \le 15$, $-19 \le l \le 19$	
Reflections collected / unique	47390 / 8042 [R_{int} = 0.0754]	
Completeness	100.0%	
Absorption correction	Semi-empirical from equivalents	
Max. and min. transmission	0.7536 and 0.5966	
Refinement method	Full-matrix least-squares on F^2	
Data / restraints / parameters	8042 / 0 / 543	
Goodness-of-fit on F^2	1.044	
Final R indices [$I > 2\sigma(I)$]	R_1 = 0.0675, wR_2 = 0.1776	
R indices (all data)	R_1 = 0.0861, wR_2 = 0.1923	
Largest diff. peak and hole	0.568 and −0.344 e · $Å^{-3}$	

Table S3. Crystal data and structure refinement for the mixed valence complex of **1•(1**$^{•+}$)$_3$.

CCDC Number	2018069
Empirical formula	$C_{62.43}H_{49.67}F_{4.50}Sb_{0.75}$
Moiety formula	4($C_{54}H_{30}$), 3(SbF_6), 1.405(C_6H_{14})
Formula weight	976.55
Temperature	100(2) K
Wavelength	1.54178 Å
Crystal system	Trigonal
Space group	$R\bar{3}c$
Unit cell dimensions	a = 48.357(2) Å α = 90°
	b = 48.357(2) Å β = 90°
	c = 45.319(2) Å γ = 120°
Volume	91777(9) Å^3
Z	72
Calculated density	1.272 Mg/m^3
Absorption coefficient	3.708 mm^{-1}
F(000)	36212.2
Crystal size	0.47 × 0.03 × 0.03 mm^3
Theta range for data collection	2.217 to 72.934°
Index ranges	$-59 \le h \le 58$, $-48 \le k \le 59$, $-56 \le l \le 45$
Reflections collected	249650
Independent reflections	20216 [R_{int} = 0.1750]
Completeness	100.0%
Absorption correction	Semi-empirical from equivalents
Max. and min. transmission	0.7536 and 0.5264
Refinement method	Full-matrix least-squares on F^2
Data / restraints / parameters	20216 / 1285 / 1393
Goodness-of-fit on F^2	1.280
Final R indices [$I > 2\sigma(I)$]	R_1 = 0.0867, wR_2 = 0.2087
R indices (all data)	R_1 = 0.1274, wR_2 = 0.2307
Largest diff. peak and hole	0.786 and −0.688 e · Å^{-3}

Table S4. Crystal data and structure refinement for the radical cation $\mathbf{1}^{\bullet+}$.[a]

CCDC Number	2018070
Empirical formula	$C_{54}H_{30}F_6Sb$
Moiety formula	$C_{54}H_{30}F_6Sb$
Formula weight	914.53
Temperature	100(2) K
Wavelength	1.54178 Å
Crystal system	Triclinic
Space group	$P\bar{1}$
Unit cell dimensions	a = 16.2193(13) Å α = 97.717(5)°
	b = 16.2807(13) Å β = 113.028(5)°
	c = 19.6549(16) Å γ = 108.551(5)°
Volume	4324.6(6) Å^3
Z	4
Calculated density	1.405 Mg/m^3
Absorption coefficient	5.583 mm^{-1}
F(000)	1836
Crystal size	0.070 × 0.060 × 0.040 mm^3
Theta range for data collection	2.558 to 68.498°
Limiting indices	$-19 \le h \le 19$, $-19 \le k \le 19$, $-23 \le l \le 23$
Reflections collected / unique	74683 [R_{int} = 0.1471]
Completeness to theta = 67.679°	98.9%
Absorption correction	Semi-empirical from equivalents
Max. and min. transmission	0.753052 and 0.585023
Refinement method	Full-matrix least-squares on F^2
Data / restraints / parameters	74683 / 198 / 1160
Goodness-of-fit on F^2	1.218
Final R indices [$I > 2\sigma(I)$]	R_1 = 0.1456, wR_2 = 0.2974
R indices (all data)	R_1 = 0.2134, wR_2 = 0.3293
Largest diff. peak and hole	1.789 and −1.649 e · Å^{-3}

[a] Disordered solvent molecules were omitted by SQUEEZE protocol[7] implemented in the PLATON software package[8].

Table S5. Crystal data and structure refinement for the dication $\mathbf{1}^{2+}$.

CCDC Number	2018071	
Empirical formula	$C_{56}H_{34}Cl_4F_{12}Sb_2$	
Moiety formula	$C_{54}H_{30}$, 2(SbF_6), 2(CH_2Cl_2)	
Formula weight	1320.13	
Temperature	100(2) K	
Wavelength	1.54178 Å	
Crystal system	Triclinic	
Space group	$P\bar{1}$	
Unit cell dimensions	*a* = 9.3884(7) Å	α = 68.376(3)°
	b = 11.5944(8) Å	β = 75.432(3)°
	c = 12.2472(9) Å	γ = 76.289(3)°
Volume	1183.94(15) $Å^3$	
Z	1	
Calculated density	1.852 Mg/m^3	
Absorption coefficient	11.909 mm^{-1}	
F(000)	648	
Crystal size	0.091 × 0.065 × 0.026 mm^3	
Theta range for data collection	3.948 to 72.508°	
Index ranges	$-11 \le h \le 11$, $-12 \le k \le 14$, $-15 \le l \le 15$	
Reflections collected	4557	
Completeness	98.4%	
Absorption correction	Semi-empirical from equivalents	
Max. and min. transmission	0.7536 and 0.5751	
Refinement method	Full-matrix least-squares on F^2	
Data / restraints / parameters	4557 / 0 / 335	
Goodness-of-fit on F^2	1.040	
Final *R* indices [$I > 2\sigma(I)$]	R_1 = 0.0546, wR_2 = 0.1351	
R indices (all data)	R_1 = 0.0590, wR_2 = 0.1397	
Largest diff. peak and hole	1.248 and −0.829 e · $Å^{-3}$	

a) Crystal unit cell of **1** (left) and **1•(1**$^{\bullet+}$)$_3$ (right):

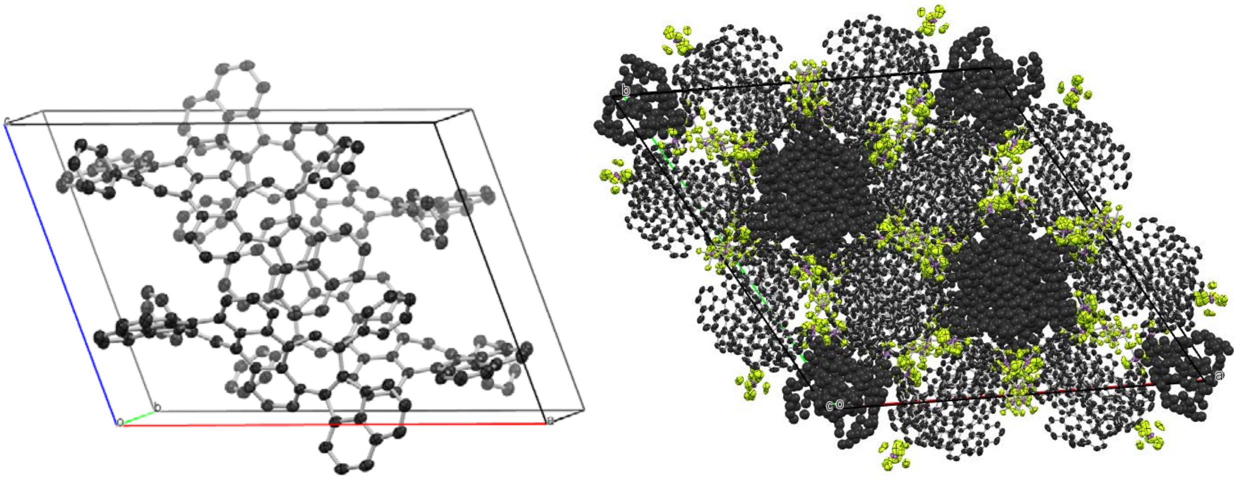

b) Crystal unit cell of **1**$^{\bullet+}$ (left) and **1**$^{2+}$ (right):

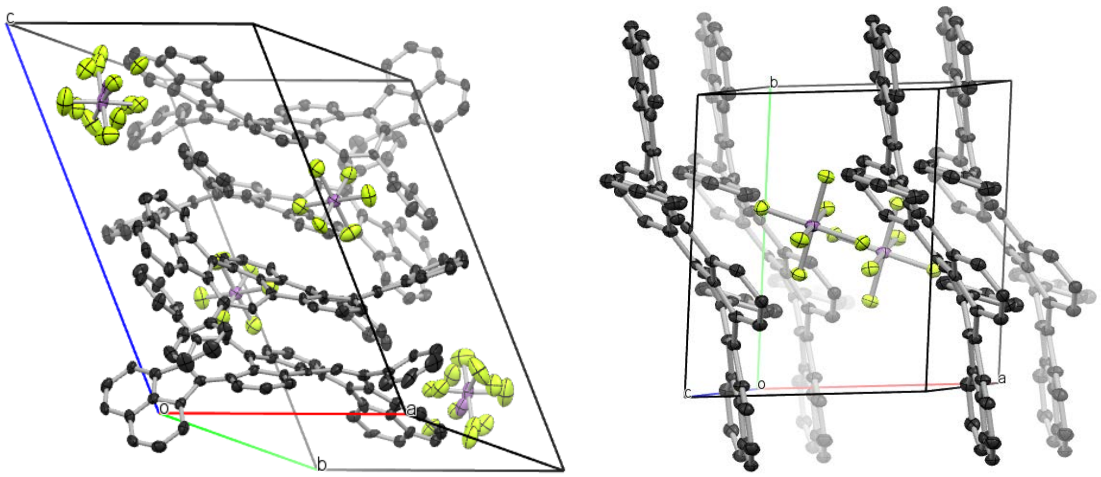

Figure S6. The crystal unit cells of neutral species **1**, the mixed valence complex of **1•(1**$^{\bullet+}$)$_3$ (with the disordered hexane solvent molecules), radical cation **1**$^{\bullet+}$ and dication species **1**$^{2+}$.

Bond lengths analysis

Neutral species **1**:

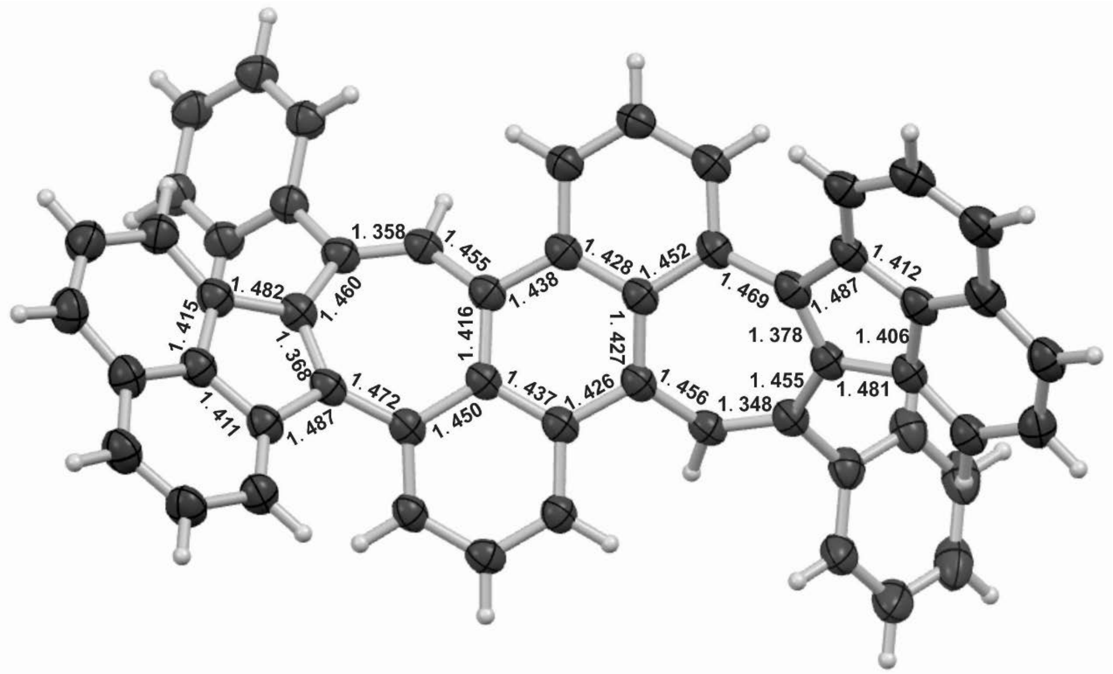

The mixed valence complex of **1•(1$^{•+}$)$_3$**:

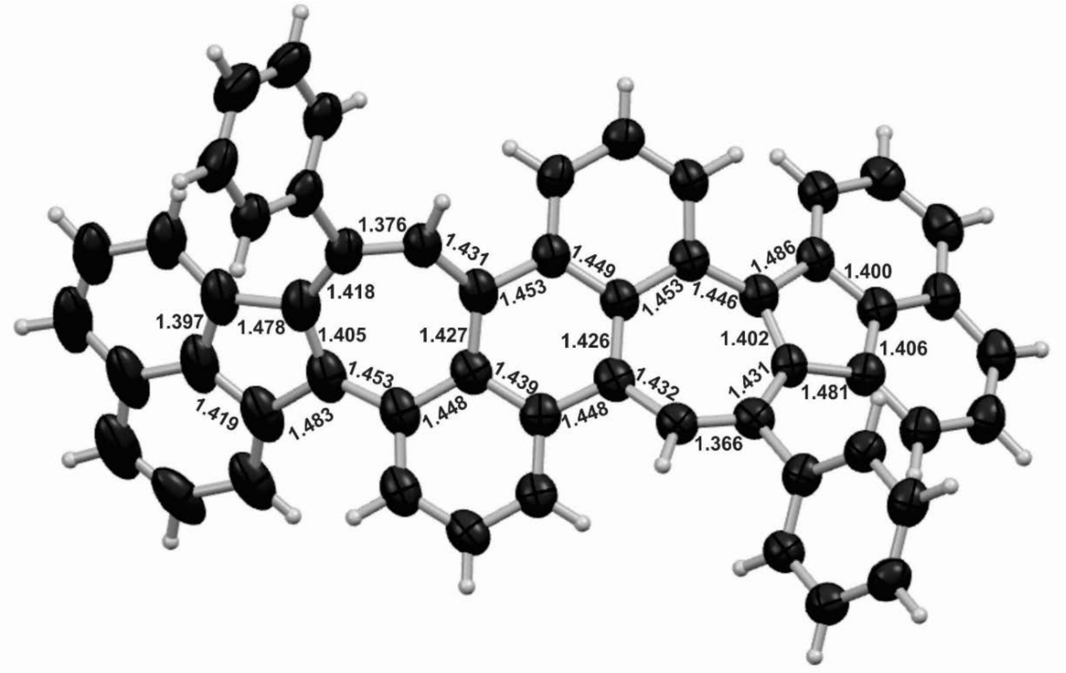

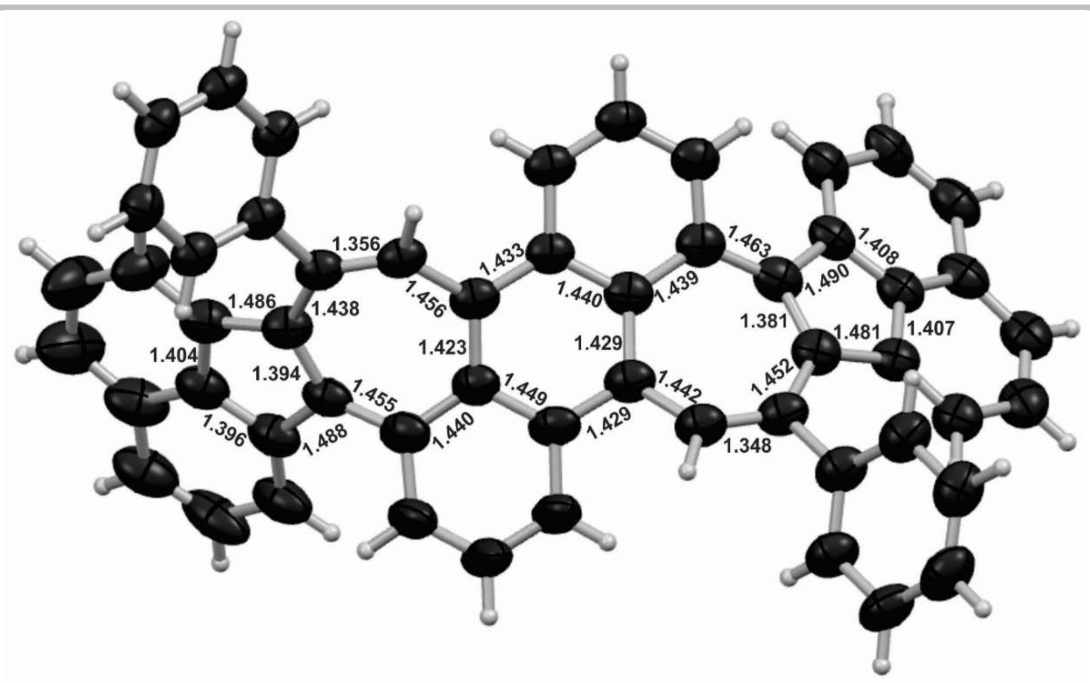

Radical cation **1**$^{•+}$:

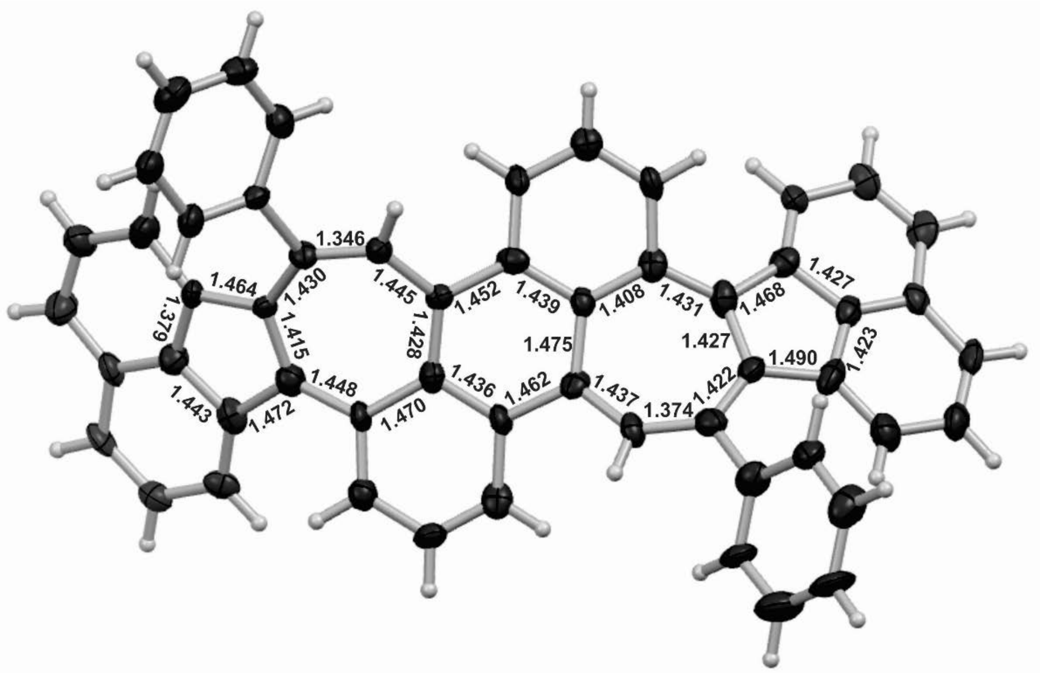

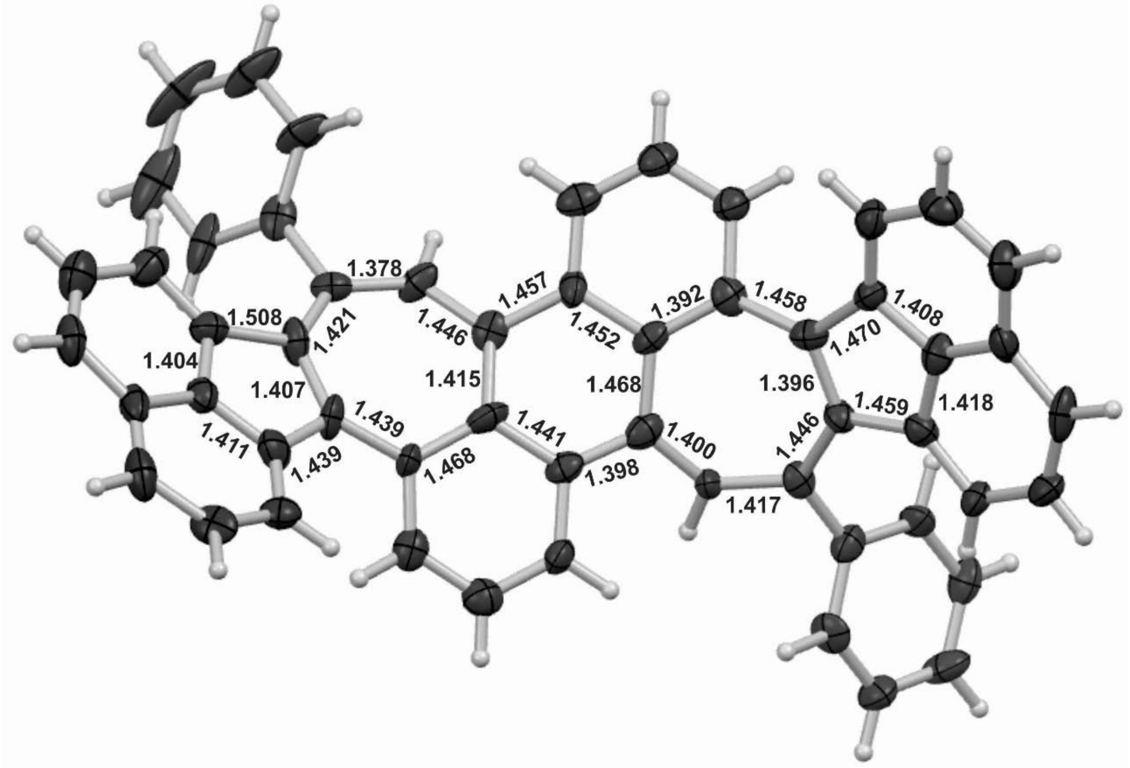

Dication 1^{2+}:

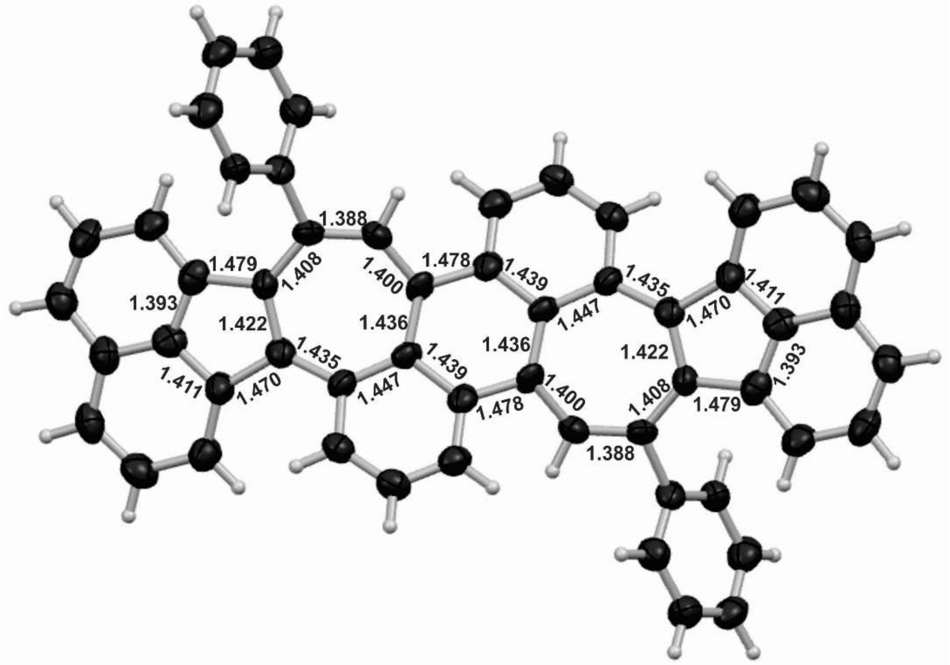

Figure S7. Selected bond lengths of neutral species **1,** the mixed valence complex of **1•(1$^{•+}$)$_3$**, radical cation **1$^{•+}$**, and dication **1**$^{2+}$, measured from their single crystal structures.

Table S6. The bond lengths (Å) of backbone of neutral species **1,** the mixed valence complex of **1•(1$^{\bullet+}$)$_3$**, radical cation **1**$^{\bullet+}$, and dication **1**$^{2+}$ obtained from their crystallographic data.

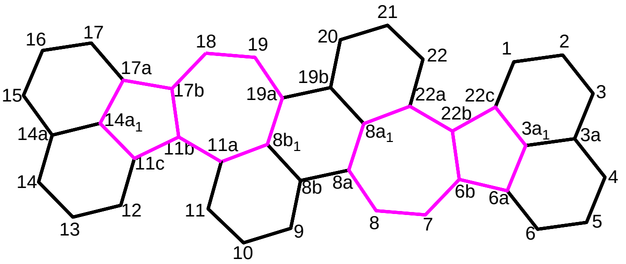

Bond	**1**	**1•(1$^{\bullet+}$)$_3$ (MV$_{II}$)**	**1•(1$^{\bullet+}$)$_3$ (MV$_{I}$)**	**1$^{\bullet+}$**		**1^{2+}**
C1-C2	1.420 (4)	1.429 (8)	1.417 (10)	1.410 (20)	1.395 (20)	1.410(10)
C2-C3	1.367 (4)	1.373 (10)	1.363 (10)	1.360 (20)	1.375 (20)	1.397(11)
C3-C3a	1.426 (4)	1.416 (10)	1.433 (9)	1.395 (20)	1.415 (25)	1.421(10)
C3a-C3a$_1$	1.394 (4)	1.395 (7)	1.392 (8)	1.380 (20)	1.390 (20)	1.399(9)
C3a$_1$-C6a	1.411 (4)	1.402 (8)	1.406 (8)	1.400 (20)	1.410 (20)	1.393(9)
C3a$_1$-C22c	1.412 (4)	1.409 (8)	1.402 (8)	1.435 (20)	1.410 (20)	1.411(10)
C3a-C4	1.417 (4)	1.416 (10)	1.400 (10)	1.420 (20)	1.425 (20)	1.413(10)
C4-C5	1.376 (4)	1.371 (10)	1.371 (9)	1.355 (20)	1.350 (20)	1.376(11)
C5-C6	1.420 (4)	1.421 (8)	1.414 (9)	1.425 (20)	1.420 (20)	1.413(10)
C6-C6a	1.377 (4)	1.380 (8)	1.378 (8)	1.390 (20)	1.365 (20)	1.390(10)
C6a-C6b	1.482 (4)	1.480 (7)	1.484 (7)	1.478 (19)	1.485 (2)	1.479(8)
C6b-C22b	1.373 (4)	1.404 (8)	1.388 (7)	1.420 (20)	1.405 (20)	1.423(9)
C6b-C7	1.458 (3)	1.425 (7)	1.445 (7)	1.425 (19)	1.435 (20)	1.408(8)
C7-C8	1.353 (3)	1.372 (7)	1.351 (7)	1.358 (19)	1.399 (19)	1.388(9)
C8-C8a	1.455 (4)	1.432 (7)	1.449 (7)	1.443 (19)	1.425 (20)	1.400(9)
C8a-C8b	1.432 (3)	1.451 (7)	1.431 (8)	1.457 (18)	1.430 (20)	1.478(9)
C8a-C8a$_1$	1.422 (3)	1.426 (7)	1.426 (7)	1.455 (20)	1.445 (20)	1.436(9)
C8a$_1$-C19b	1.433 (3)	1.445 (7)	1.445 (7)	1.438 (19)	1.445 (19)	1.439(9)
C8a$_1$-C22a	1.451 (3)	1.450 (7)	1.440 (7)	1.440 (18)	1.430 (20)	1.447(9)
C19b-C20	1.426 (3)	1.396 (7)	1.421 (7)	1.425 (20)	1.405 (20)	1.382(10)
C20-C21	1.365 (3)	1.379 (7)	1.362 (9)	1.368 (19)	1.385 (20)	1.379(10)
C21-C22	1.405 (4)	1.393 (8)	1.391 (8)	1.375 (20)	1.370 (20)	1.371(10)
C22-C22a	1.382 (4)	1.391 (8)	1.389 (7)	1.405 (20)	1.420 (20)	1.410(9)
C22a-C22b	1.471 (4)	1.450 (7)	1.459 (8)	1.440 (20)	1.450 (20)	1.435(9)
C22b-C22c	1.487 (3)	1.485 (7)	1.489 (7)	1.470 (20)	1.455 (20)	1.470(8)
C1-C22c	1.375 (4)	1.389 (8)	1.392 (8)	1.370 (20)	1.400 (20)	1.397(9)

Table S7. The symmetry-averaged bond lengths (Å) of neutral species **1,** the mixed valence complex of **1•(1**$^{\bullet+}$**)**$_3$, radical cation **1**$^{\bullet+}$ and dication **1**$^{2+}$ Experimental values were obtained according to the values from Table S6. DFT-calculated bond lengths ((U)B3LYP/6-31+G(d)) were taken from the optimized structures in Tables S16–S21.

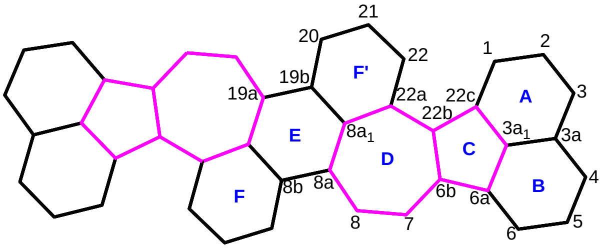

Bond	Ring	1			1•(1$^{\bullet+}$)$_3$	1$^{\bullet+}$			1^{2+}		
		Exp.	*Calc.(cis)*	*Calc.(trans)*	*Exp.*	*Exp.*	*Calc.(cis)*	*Calc.(trans)*	*Exp.*	*Calc.(cis)*	*Calc.(trans)*
C1-C2	A	1.420 (4)	1.425	1.425	1.423(9)	1.403(20)	1.418	1.419	1.410(10)	1.410	1.411
C2-C3	A	1.367 (4)	1.386	1.386	1.368(10)	1.368(20)	1.388	1.389	1.397(11)	1.395	1.395
C3-C3a	A	1.426 (4)	1.425	1.425	1.425(10)	1.405(22)	1.423	1.423	1.421(10)	1.419	1.419
C3a-C3a$_1$	A, B	1.394 (4)	1.402	1.402	1.394(7)	1.385(20)	1.404	1.404	1.399(9)	1.407	1.406
C3a$_1$-C6a	B, C	1.411 (4)	1.414	1.414	1.404(8)	1.405(20)	1.412	1.412	1.393(9)	1.409	1.410
C3a$_1$-C22c	A, C	1.412 (4)	1.416	1.416	1.406(8)	1.423(20)	1.416	1.416	1.411(10)	1.418	1.418
C3a-C4	B	1.417 (4)	1.426	1.426	1.408(10)	1.423(20)	1.424	1.424	1.413(10)	1.423	1.423
C4-C5	B	1.376 (4)	1.386	1.386	1.371(9)	1.353(20)	1.386	1.387	1.376(11)	1.387	1.388
C5-C6	B	1.420 (4)	1.425	1.425	1.418(8)	1.423(20)	1.422	1.421	1.413(10)	1.419	1.419
C6-C6a	B	1.377 (4)	1.384	1.385	1.379(8)	1.378(20)	1.388	1.388	1.390(10)	1.392	1.392
C6a-C6b	C	1.482 (4)	1.483	1.482	1.482(7)	1.482(19)	1.479	1.478	1.479(8)	1.478	1.475
C6b-C22b	C, D	1.373 (4)	1.387	1.387	1.396(7)	1.413(19)	1.411	1.408	1.423(9)	1.440	1.434
C6b-C7	D	1.458 (3)	1.454	1.452	1.435(7)	1.430(19)	1.433	1.432	1.408(8)	1.412	1.412
C7-C8	D	1.353 (3)	1.364	1.363	1.362(7)	1.379(19)	1.387	1.385	1.388(9)	1.417	1.411
C8-C8a	D	1.455 (4)	1.461	1.460	1.441(7)	1.434(19)	1.426	1.431	1.400(9)	1.392	1.402
C8a-C8b	E	1.432 (3)	1.429	1.434	1.441(7)	1.444(19)	1.453	1.456	1.478(9)	1.478	1.478
C8a-C8a$_1$	D, E	1.422 (3)	1.424	1.424	1.426(7)	1.450(20)	1.435	1.432	1.436(9)	1.442	1.440
C8a$_1$-C19b	E, F′	1.433 (3)	1.446	1.442	1.445(7)	1.442(19)	1.440	1.438	1.439(9)	1.431	1.435
C8a$_1$-C22a	D, F′	1.451 (3)	1.451	1.456	1.445(7)	1.435(19)	1.442	1.448	1.447(9)	1.437	1.411
C19b-C20	F′	1.426 (3)	1.427	1.430	1.409(7)	1.415(20)	1.411	1.414	1.382(10)	1.397	1.388
C20-C21	F′	1.365 (3)	1.373	1.372	1.371(8)	1.377(19)	1.386	1.384	1.379(10)	1.398	1.396
C21-C22	F′	1.405 (4)	1.410	1.411	1.392(8)	1.373(20)	1.383	1.394	1.371(10)	1.382	1.381
C22-C22a	F′	1.382 (4)	1.388	1.387	1.390(7)	1.413(20)	1.406	1.404	1.410(9)	1.419	1.418
C22a-C22b	D	1.471 (4)	1.469	1.470	1.455(7)	1.445(20)	1.456	1.456	1.435(9)	1.446	1.445
C22b-C22c	C	1.487 (3)	1.483	1.485	1.487(7)	1.463(20)	1.472	1.473	1.470(8)	1.454	1.456
C1-C22c	A	1.375 (4)	1.386	1.386	1.391(8)	1.385(20)	1.391	1.391	1.397(9)	1.400	1.399

Analysis of bond lengths of curved non-alternant PAH **1** shows that, upon oxidation, the bond length of the ethylene bridge of acenaphthene moiety (C6b-C22b) becomes longer from 1.373(4) Å for the neutral species **1** to 1.423(9) Å for the dication species **1**$^{2+}$. This corresponds to a change from localized C–C double bond to delocalized C–C aromatic bond (-C_{Ar}≃C_{Ar}-, 1.384 Å; -C_{Ar}–C_{Ar}-, 1.487

Å).[10], [11] Notably, this change is systematic as the oxidation state increases: the lengths of bond C6b-C22b for **1**•(**1**$^{\bullet+}$)$_3$ and **1**$^{\bullet+}$ are 1.396(7) and 1.413(19) Å, respectively. The change of the length of bond C7-C8 in the seven-membered ring is from 1.353(3) Å for **1** to 1.388(9) Å for **1**$^{2+}$. This indicates a shift from a C–C double bond (-C=C-, 1.331 Å) to an aromatic bond (-C$_{Ar}$≃C$_{Ar}$-, 1.384 Å).[10] The bond C8b-C8b becomes longer from 1.432(3) Å for **1** to 1.478(9) for **1**$^{2+}$. This can be considered as a shift from an equivalent bond of anthracene (-C9≃C9a-, 1.400 Å) to a single bond between two aromatic carbons (-C$_{Ar}$–C$_{Ar}$-, 1.487 Å).[10] Moreover, it's worth mentioning that the lengths of bond C6b-C22b, C7-C8, C8a-C8b for **1**•(**1**$^{\bullet+}$)$_3$ lie between those for **1** and **1**$^{\bullet+}$, thus further supporting the existence of this intermolecular MV species **1**•(**1**$^{\bullet+}$)$_3$. These observations suggest aromatization of the seven-membered ring as well as dearomatization of the central benzene ring. The former can be further corroborated by calculated HOMA indices (Figure S8) and observed planarization of the seven-membered ring, where the deviation of the mean plane decreased from 0.190 to 0.145 Å (Figure S9) and the torsion angle reduced from 39.5° to 26.9°. Additionally, the calculated bond lengths of *cisoid* and *transoid* of neutral species **1**, radical cation **1**$^{\bullet+}$ and dication **1**$^{2+}$ are nearly identical, and they are also comparable to those measured from their crystal structures.

Table S8. Calculated HOMA values of neutral species **1,** the mixed valence species **1•(1$^{\bullet+}$)$_3$**, radical cation **1**$^{+\bullet}$, and dication **1**$^{2+}$ based on their crystal structures.[12]

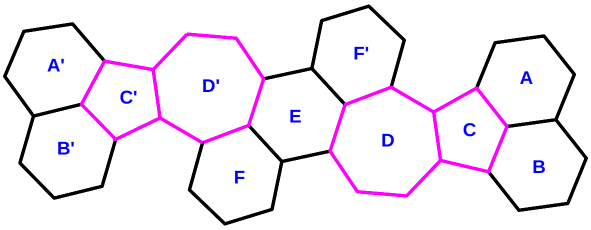

Rings	A′	B′	C′	D′	F	E	F′	D	C	B	A
**1**	0.85	0.83	-0.05	0.15	0.62	0.58	0.66	0.17	0.01	0.91	0.85
**1•(1$^{\bullet+}$)$_3$**	0.90	0.87	0	0.42	0.67	0.38	0.68	0.46	0.01	0.92	0.82
**1**$^{+\bullet}$	0.89	0.80	0.32	0.42	0.66	0.20	0.56	0.46	0.07	0.66	0.89
**1**$^{2+}$	0.93	0.89	0.14	0.64	0.70	-0.11	0.71	0.64	0.13	0.94	0.89

HOMA values were calculated by using the following equation on the basis of their X-ray crystallographic data:

$$\text{HOMA} = 1 - \frac{\alpha}{n}\,\boldsymbol{\Sigma}\,(R_{apt} - R_i)^2$$

n is the number of bonds taken into the summation, *α* is an empirical constant of 257.7, R_{apt} is an optimal bond length of 1.388 Å and R_i is the individual bond length of the ring.

Table S9. Nonplanarity of selected rings of neutral species **1**, the mixed valence species **1•(1**$^{\bullet+}$)$_3$, radical cation **1**$^{\bullet+}$ and dication **1**$^{2+}$ according to crystal structures.

Ring / Compound	D'	E	D
**1**	0.182	0.085	0.198
**1•(1$^{\bullet+}$)$_3$**	0.163	0.077	0.161
**1**$^{\bullet+}$	0.159	0.079	0.172
**1**$^{2+}$	0.145	0.045	0.145

Nonplanarity values were calculated by constructing a best-fit plane of the individual rings for the molecular structures found in the crystals and measuring the distance of each carbon atom of the ring from this plane, and then taking the avarage values.[13]

DFT-computational Studies and Theoretical Analyses[14]

1) NICS(1)$_{zz}$ calculations[15]

Table S10. NICS(1)$_{zz}$ values of neutral species **1,** radical cation $\mathbf{1}^{\bullet+}$ and dication $\mathbf{1}^{2+}$ were calculated at GIAO-B3LYP/6-31G(d) level of theory using single crystal structures.

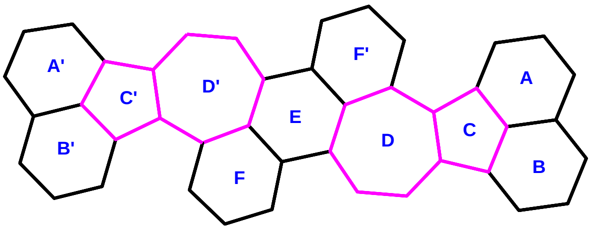

Ring	NICS (n)	A'	B'	C'	D'	E	F'	F	C	D	B	A
**1**	**−1**	-23.83	-22.33	8.52	18.48	-17.72	-20.55	-19.64	7.62	24.15	-24.03	-23.53
	**1**	-22.80	-21.95	6.49	15.49	-24.95	-17.97	-17.32	4.92	12.55	-22.64	-21.46
	Ave.	-23.32	-22.14	7.50	16.98	-20.34	-19.26	-18.48	6.28	18.35	-23.34	-22.50
$\mathbf{1}^{\bullet+}$	**−1**	-23.10	-23.71	1.23	10.07	-3.16	-17.72	-20.14	1.39	6.70	-23.20	-23.36
	**1**	-20.43	-21.77	0.93	3.21	-6.75	-16.87	-20.33	1.83	6.39	-20.65	-21.84
	Ave.	-21.77	-22.74	1.08	6.64	-4.96	-17.30	-20.24	1.61	6.55	-21.93	-22.60
	**−1**	-22.25	-22.82	1.13	3.67	-2.85	-19.83	-17.0	3.03	2.31	-22.59	-21.92
	**1**	-21.12	-20.68	0.88	5.61	-6.26	-18.47	-17.42	2.54	5.58	-21.71	-21.15
	Ave.	-21.69	-21.75	1.00	4.64	-4.56	-19.15	-17.21	2.79	3.95	-22.15	-21.54
	Ave.	-21.73	-22.25	1.04	5.64	-4.67	-18.23	-18.73	2.20	5.25	-22.04	-22.07
$\mathbf{1}^{2+}$	**−1**	-22.40	-22.41	-5.15	-4.56	11.25	-21.97	-18.70	-5.16	-5.14	-22.41	-22.40
	**1**	-20.76	-20.34	-2.76	-4.16	11.25	-18.70	-21.97	-2.76	-4.26	-20.76	-20.34
	Ave.	-21.58	-21.38	-3.96	-4.36	11.25	-20.33	-20.34	-3.96	-4.70	-21.59	-21.37

NICS(−1) and NICs(1) indices represent the positions where are 1 Å below and above the ring centre, respectively. NICS$_{Ave.}$ was calculated according to NICS(−1) and NICS(1) values using the formula: NICS$_{Ave.}$ = $\frac{\text{NICS}(-1)+\text{NICS}(1)}{2}$.

Table S11. NICS(1)$_{zz}$ values of C_2-symmetric *cisoid* and C_i-symmetric *transoid* neutral species **1,** radical cation $\mathbf{1}^{\bullet+}$ and dication $\mathbf{1}^{2+}$ calculated at GIAO-B3LYP/6-31+G(d) level of theory using the opimized structures at the same level of theory.

Compounds	Conformation	NICS (n)	A	B	C	D	E	F
**1**	*cisoid*	**1**	-22.85	-21.7	6.43	14.53	-21.7	-19.55
	transoid	**1**	-22.83	-22.1	6.43	14.06	-21.42	-18.00
$\mathbf{1}^{\bullet+}$	*cisoid*	**1**	-22.05	-21.32	1.40	5.49	-5.24	-19.89
	transoid	**1**	-21.95	-21.43	1.52	4.43	-5.93	-20.48
$\mathbf{1}^{2+}$	*cisoid*	**1**	-21.25	-20.75	-4.18	-6.00	9.12	-22.06
	transoid	**1**	-21.07	-20.93	-3.85	-6.10	10.37	-21.11

The calculated NICS(1)$_{zz}$ values in Table S11 suggest that the optimized *cisoid* and *transoid* conformations of neutral species **1**, radical cation $\mathbf{1}^{\bullet+}$ and dication $\mathbf{1}^{2+}$ have comparable magnetic aromaticity. These NICS(1)$_{zz}$ values based on the energy-minimized structures are also similar to those calculated from their crystal structures (Table S10).

2) Mulliken charges

Table S12. DFT calculated carbon-atomic Mulliken charges of the heptagons of neutral species **1**, radical cation **1**$^{•+}$ and dication **1**$^{2+}$ at (U)B3LYP/6-31+G(d) level of theory based on their crystal structures.

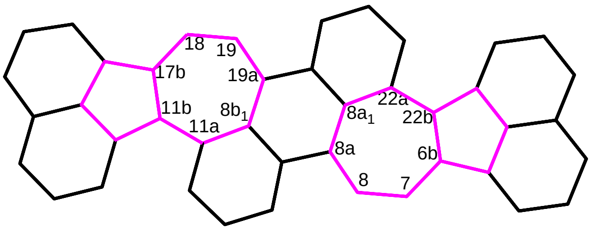

Atoms / Compound	**1**	**1**$^{•+}$	**1**$^{2+}$
**C7**	-0.476999	-0.175080	0.168990
**C8**	-1.050229	-1.151040	-0.607744
**C8a**	1.082638	1.017030	0.493236
**C8a$_1$**	0.561414	0.551371	0.435710
**C22a**	0.590803	0.794783	0.845380
**C22b**	-0.021105	0.077999	0.023270
**C6b**	0.045665	0.079643	0.304053
**C11a**	0.502301	0.397255	0.845064
**C11b**	-0.006014	0.005872	0.023220
**C17b**	0.047547	0.122690	0.303857
**C18**	-0.498894	-0.212390	0.168839
**C19**	-1.119314	-1.190143	-0.607445
**C19a**	0.995265	0.799517	0.492518
**C8b$_1$**	0.622156	0.291667	0.435778

Compared with those in neutral species **1** and radical cation **1**$^{•+}$, the positive charges mainly locate on the heptagonal rings in dication **1**$^{2+}$, suggesting the formation of tropylium-like cations. This result is in line with the observation in the crystal structures.

3) Electrostatic potential (ESP) map

Neutral species **1**:

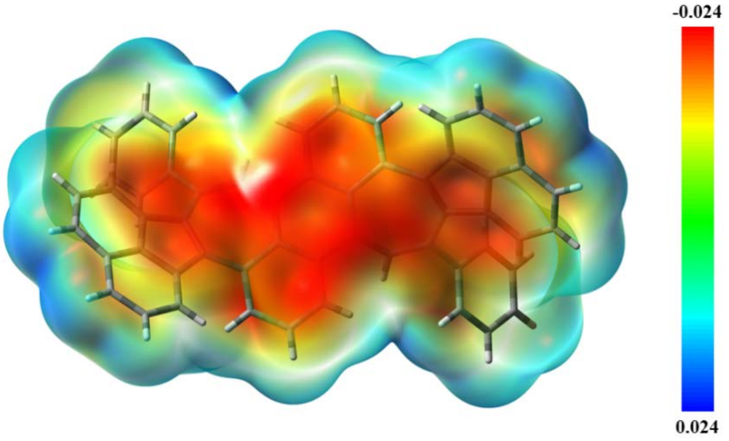

Radical cation $\mathbf{1}^{\bullet+}$:

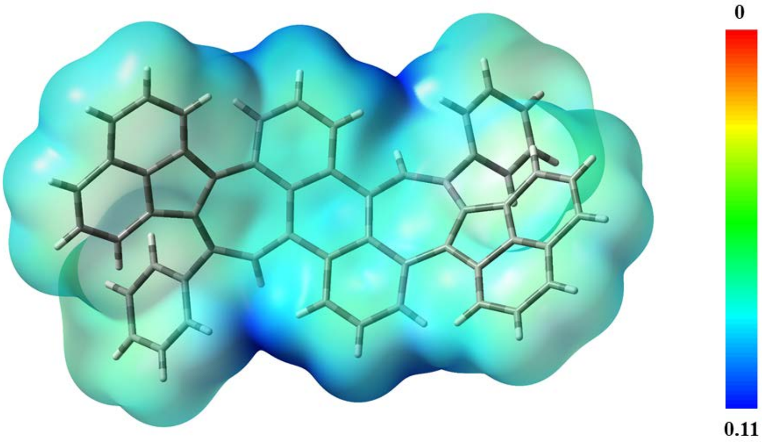

Dication $\mathbf{1}^{2+}$:

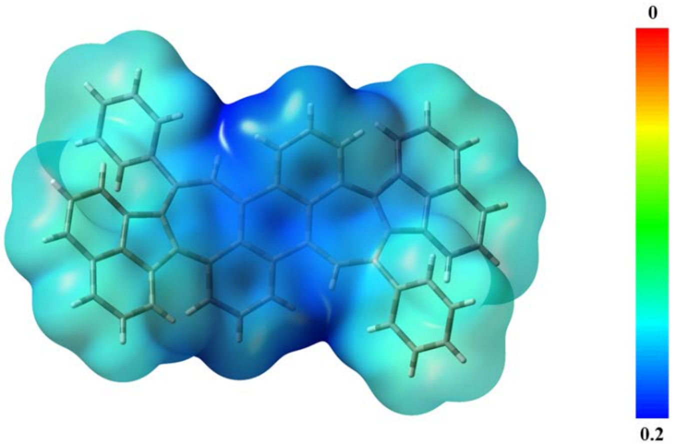

Figure S8. DFT calculated electrostatic potential (ESP) map of neutral species **1** (top), radical cation $\mathbf{1}^{\bullet+}$ (middle) and dication $\mathbf{1}^{2+}$ (bottom) at (U)B3LYP/6-31+G(d) level of theory using the molecular structures obtained from crystallographic analyses. Red and blue regions represent negative and positive charges, respectively.

The ESP maps reveal that the negative charge (or electron density) mainly localizes in the heptagonal rings and central anthracene core of the backbone of neutral species **1**, suggesting the electron-rich π-system; In radical cation $\mathbf{1}^{\bullet+}$, the positive charge predominantly localizes at the periphery of the central core, as observed in the single crystal structure, in which the counterion (SbF_6^-) locates at the edge of the central core; In dication $\mathbf{1}^{2+}$, the two heptagonal rings and central anthracene core carrying positive charges further support the formation of tropylium-like cations.

4) Time-dependent DFT calculated excited states of neutral species **1** (B3LYP/6-31+G(d)), radical cation **1**$^{\bullet+}$ (UB3LYP/6-31+G(d)) and dication **1**$^{2+}$(B3LYP/6-31+G(d)).

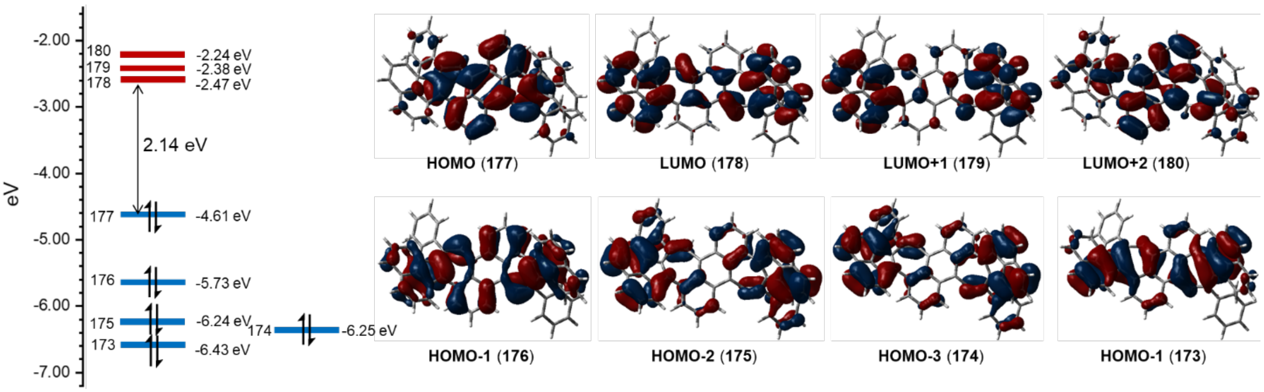

Figure S9. Plots of frontier molecular orbitals and energy diagram of neutral species **1**.

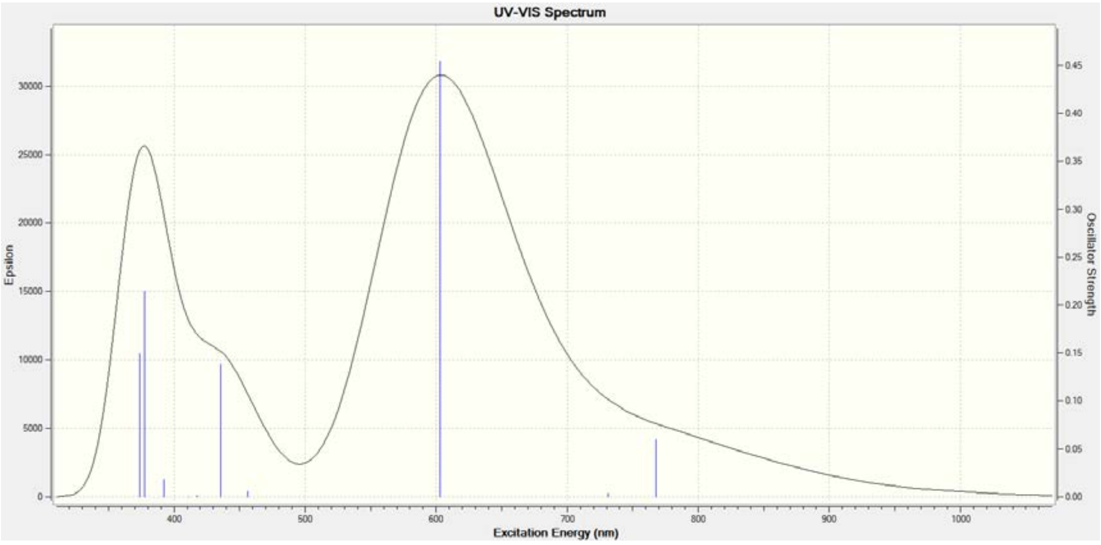

Figure S10. Time-dependent DFT calculated UV/Vis spectrum of the neutral species **1**.

Table S13. Excited states of neutral species **1** calculated by time-dependent DFT calculations.

Excited State	Main contribution	Energy (eV)	Energy (nm)	Oscillator strengths
**S1**	HOMO → LUMO (0.69457)	1.6149	767.76	0.0598
**S2**	HOMO → LUMO+1 (0.70249)	1.6958	731.14	0.0039
**S3**	HOMO → LUMO+2 (0.69420)	2.0561	603.01	0.4545
**S4**	HOMO-1 → LUMO (0.67864)<br>HOMO-1 → LUMO+2 (-0.12082)	2.7173	456.28	0.0060
**S5**	HOMO-4 → LUMO (0.12567)<br>HOMO-1 → LUMO+1 (0.64771)<br>HOMO → LUMO+3 (-0.19059)	2.8463	435.60	0.1379
**S8**	HOMO-3 → LUMO (-0.13422)<br>HOMO → LUMO+4 (0.66787)	3.1612	392.20	0.0182
**S9**	HOMO-5 → LUMO (-0.10403)<br>HOMO-5 → LUMO+2 (0.22980)<br>HOMO-3 → LUMO+1 (-0.13776)<br>HOMO-2 → LUMO (-0.31787)<br>HOMO → LUMO+5 (0.47530)<br>HOMO → LUMO+7 (0.22243)	3.2861	377.30	0.2145

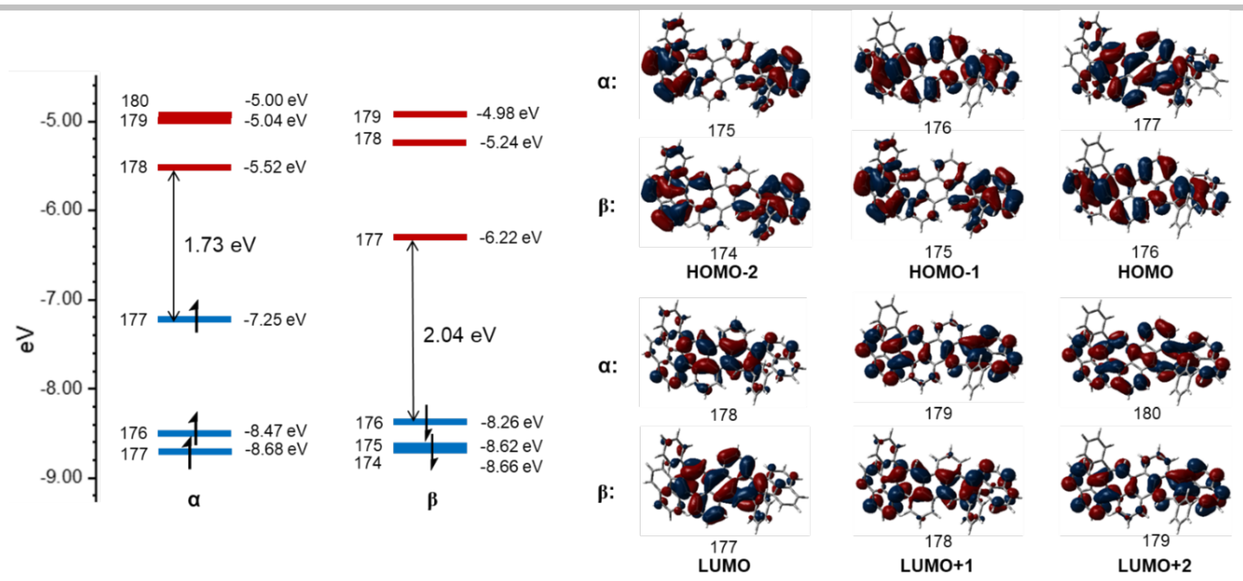

Figure S11. Plots of frontier molecular orbitals and energy diagram of radical cation $\mathbf{1}^{\bullet+}$.

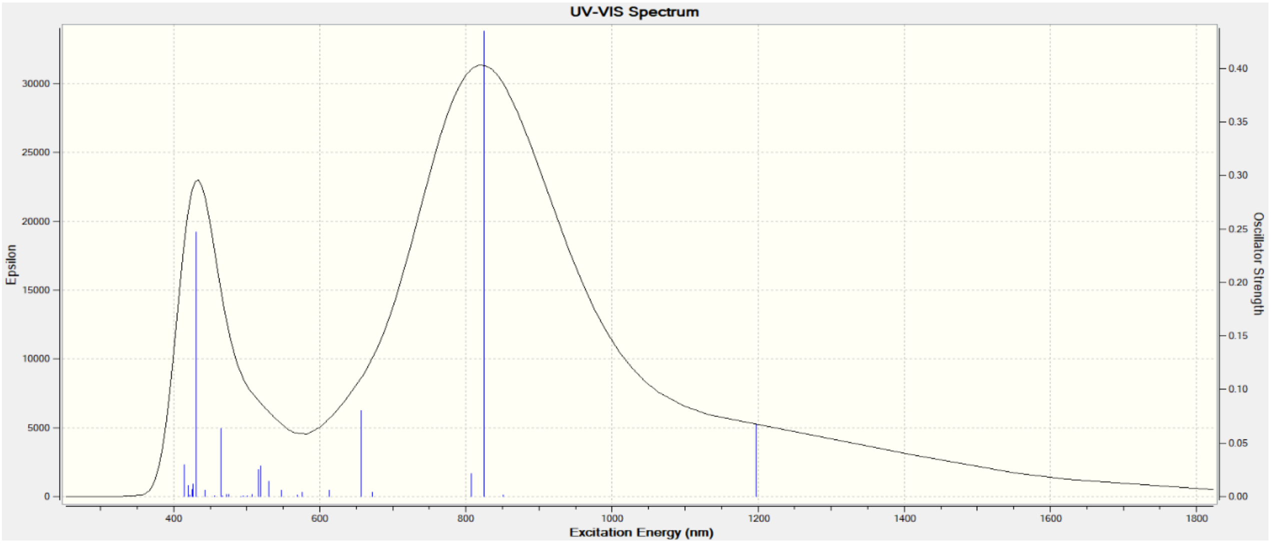

Figure S12. Time-dependent DFT calculated UV/Vis spectrum of radical cation $\mathbf{1}^{\bullet+}$.

Table S14. Excited states of radical cation $\mathbf{1}^{\bullet+}$ calculated by time-dependent DFT calculations.

Excited State	Main contribution	Energy (eV)	Energy (nm)	Oscillator strengths
**S1**	α: HOMO-1 → LUMO+1 (0.10522)<br>α: HOMO → LUMO (0.92586)<br>β: HOMO → LUMO (-0.33130)	1.0343	1198.70	0.0676
**S3**	α: HOMO → LUMO (0.32317)<br>α: HOMO → LUMO+2 (-0.33881)<br>β: HOMO → LUMO (0.84761)	1.5045	824.09	0.4342
**S4**	α: HOMO-1 → LUMO+1 (-0.14488)<br>α: HOMO → LUMO+2 (0.87965)<br>β: HOMO-2 → LUMO (-0.13242)<br>β: HOMO → LUMO (0.36709)<br>β: HOMO → LUMO+2 (-0.15899)	1.5363	807.03	0.0218
**S6**	α: HOMO → LUMO+2 (0.12884)<br>β: HOMO-2 → LUMO (0.96210)<br>β: HOMO-1 → LUMO+1 (-0.12840)	1.8876	656.83	0.0802

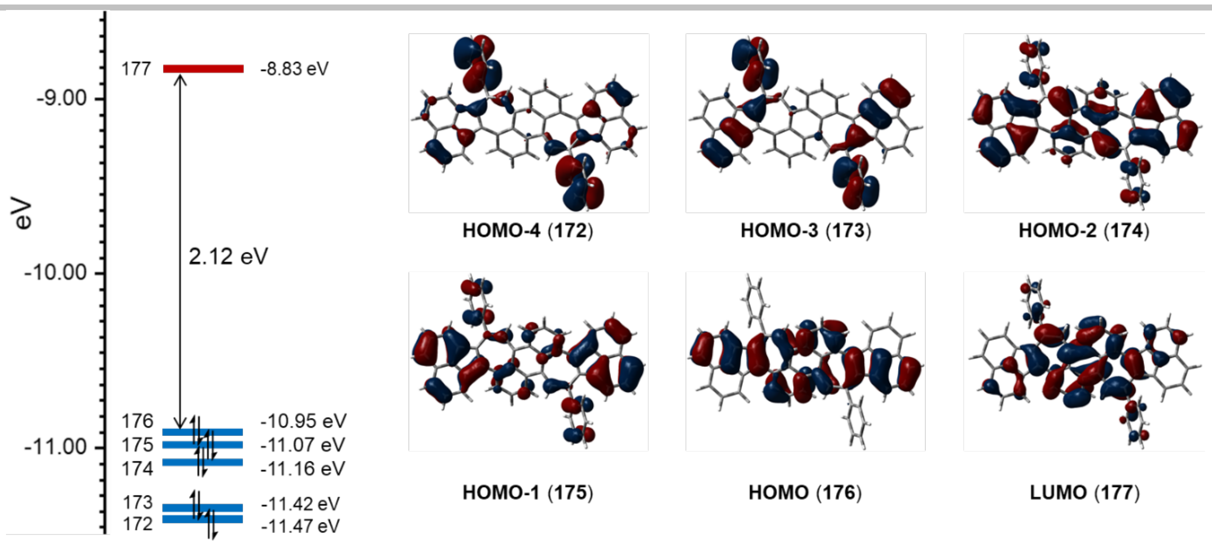

Figure S13. Plots of frontier molecular orbitals and energy diagram of dication **1**$^{2+}$.

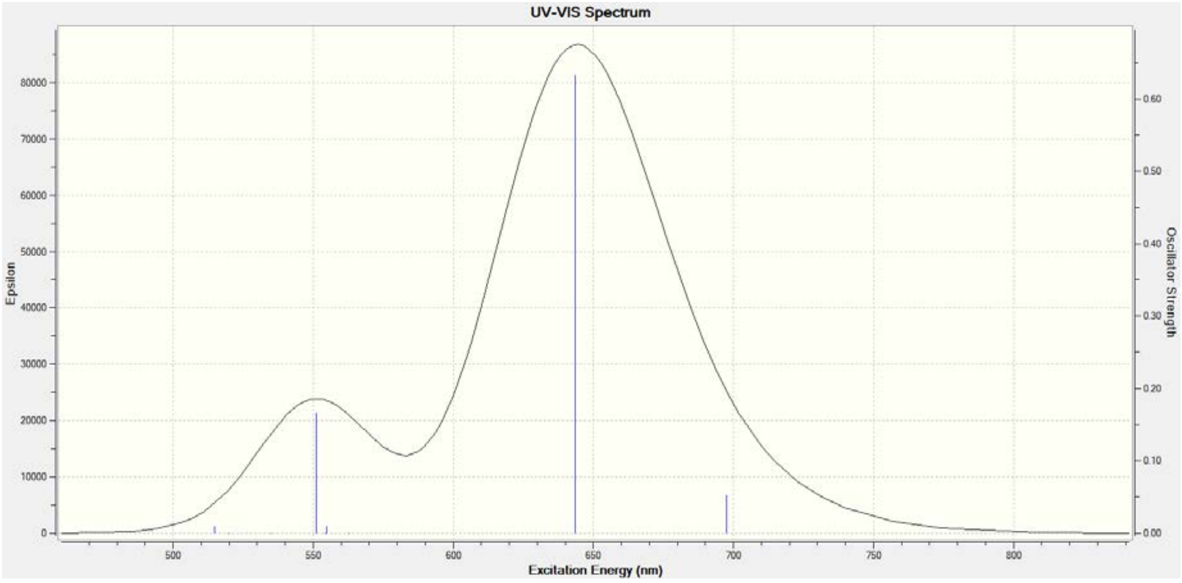

Figure S14. Time-dependent DFT calculated UV/Vis spectrum of dication **1**$^{2+}$.

Table S15. Excited states of dication **1**$^{2+}$ calculated by time-dependent DFT calculations at B3LYP/6-31+G(d) level of theory.

Excited State	Main contribution	Energy (eV)	Energy (nm)	Oscillator strengths
**S1**	HOMO-2 → LUMO (0.39158)<br>HOMO → LUMO (0.57604)	1.7771	697.69	0.0518
**S2**	HOMO-1 → LUMO (0.69515)	1.7774	697.56	0.0000
**S3**	HOMO-2 → LUMO (0.57548)<br>HOMO → LUMO (-0.39852)	1.9266	643.55	0.6322
**S7**	HOMO-4 → LUMO (0.69558)	2.2495	551.17	0.1662

Compound	HOMO	LUMO	HOMO-LUMO Gap (eV)
**1**	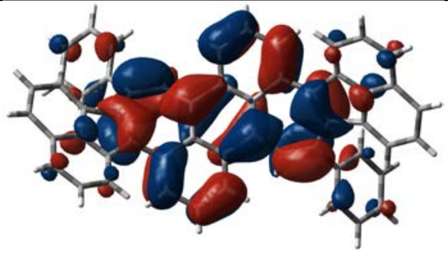 (-4.48 eV)	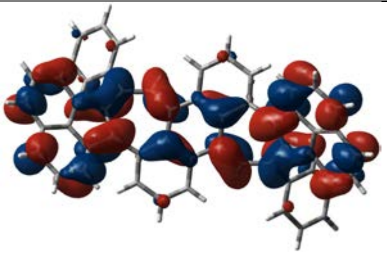 (-2.40 eV)	2.08
$\mathbf{1}^{\bullet+}$	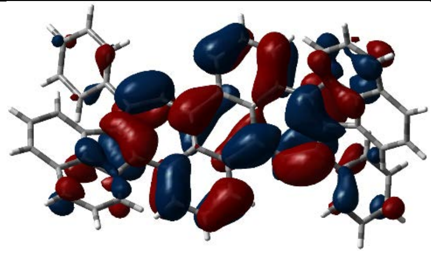 **α-SOMO** (-7.25 eV)	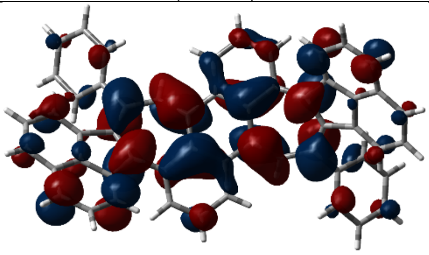 **α-LUMO** (-5.52 eV)	1.73
	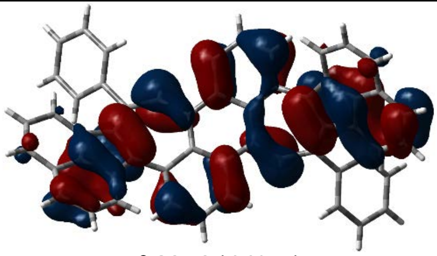 **β-SOMO** (-8.26 eV)	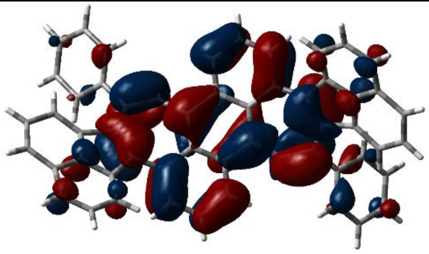 **β-LUMO** (-6.22 eV)	2.04
$\mathbf{1}^{2+}$	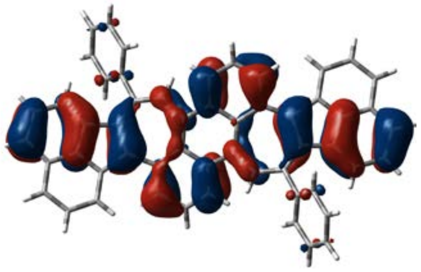 (-11.02 eV)	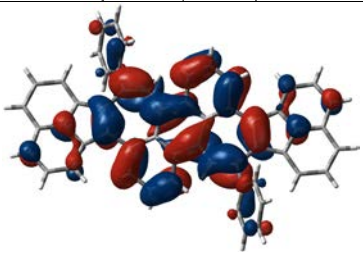 (-8.92 eV)	2.10

Figure S15. DFT calculated ((U)B3LYP/6-31+G(d)) profiles of frontier molecular orbitals of neutral species **1**, radical cation $\mathbf{1}^{\bullet+}$ and dication $\mathbf{1}^{2+}$ based on their crystal structures.

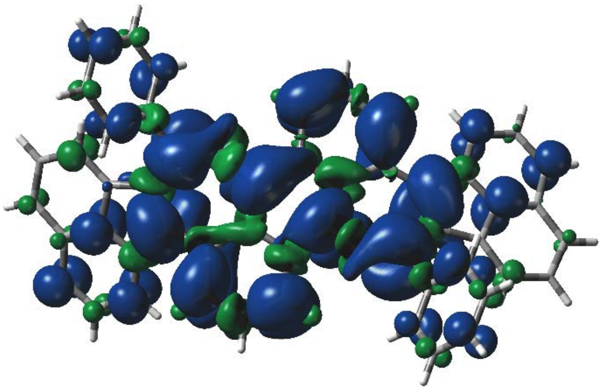

Figure S16. Spin density distribution map of radical cation $\mathbf{1}^{\bullet+}$ (iosvalue 0.0004 a.u.) calculated by using the molecular structures derived from crystallographic analyses. Blue and green colors represent α-, β-spin density, respectively.

5) Cartesian coordinates of DFT calculated geometry

Table S16. Cartesian coordinates of the optimized geometry (B3LYP/6-31+G(d)) of C_2-symmetric *cisoid* **1** and its Gibbs free energy.

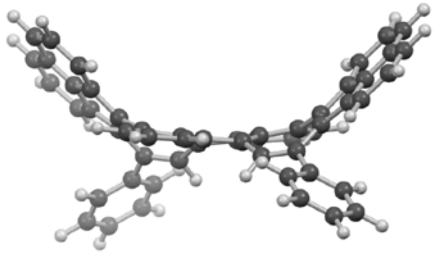

Atoms	X	Y	Z	Atoms	X	Y	Z
C	-1.1394201	3.6132083	0.3818001	C	-0.604248	-1.2942791	-0.584852
C	-1.6615521	2.3337532	-0.1182979	C	0.8004731	-1.1942291	-0.369153
C	-0.8004731	1.1942291	-0.369153	C	1.6615521	-2.3337532	-0.1182979
C	0.604248	1.2942791	-0.584852	C	3.0381072	-2.1694382	-0.1895009
C	1.2306121	2.4832702	-1.15709	H	3.6800163	-3.0319992	-0.0444859
H	2.0048671	2.2503542	-1.8852741	C	3.6234173	-0.9038871	-0.402504
C	0.9724691	3.8109383	-0.987737	H	4.7061073	-0.8094341	-0.429526
C	0.001769	4.2921243	-0.0184499	C	2.8293542	0.212352	-0.496217
C	0.097984	5.5555184	0.7516051	H	3.2913662	1.1921801	-0.540611
C	-1.0170441	5.5743834	1.6216012	C	1.4048421	0.115126	-0.478689
C	-1.8028771	4.4104243	1.4423792	C	-1.2306121	-2.4832702	-1.15709
C	-2.8953442	4.2373943	2.2768912	H	-2.0048671	-2.2503542	-1.8852741
H	-3.5206163	3.3510002	2.2291042	C	-0.9724691	-3.8109383	-0.987737
C	-3.1928452	5.2407804	3.2436423	C	-0.001769	-4.2921243	-0.0184499
H	-4.0557723	5.0958384	3.8887693	C	1.1394201	-3.6132083	0.3818001
C	-2.4198112	6.3811555	3.3947773	C	1.8028771	-4.4104243	1.4423792
H	-2.6790712	7.1210165	4.1487334	C	2.8953442	-4.2373943	2.2768912
C	-1.2713621	6.5723905	2.5718923	H	3.5206163	-3.3510002	2.2291042
C	-0.335785	7.6469416	2.6222113	C	3.1928452	-5.2407804	3.2436423
H	-0.478299	8.4601736	3.3303593	H	4.0557723	-5.0958384	3.8887693
C	0.7617101	7.6449366	1.7758492	C	2.4198112	-6.3811555	3.3947773
H	1.4739521	8.4644786	1.8293032	H	2.6790712	-7.1210165	4.1487334
C	1.0024711	6.6011065	0.8362481	C	1.2713621	-6.5723905	2.5718923
H	1.8875941	6.6481265	0.2102491	C	1.0170441	-5.5743834	1.6216012
C	1.7279761	4.7978473	-1.8143301	C	-0.097984	-5.5555184	0.7516051
C	3.1220282	4.7123053	-1.9747641	C	-1.0024711	-6.6011065	0.8362481
H	3.6738713	3.9417573	-1.442452	H	-1.8875941	-6.6481265	0.2102491
C	3.8091323	5.6199784	-2.7844141	C	-0.7617101	-7.6449366	1.7758492
H	4.8885553	5.5392644	-2.8873231	H	-1.4739521	-8.4644786	1.8293032
C	3.1146722	6.6332365	-3.4510172	C	0.335785	-7.6469416	2.6222113
H	3.6482963	7.3403985	-4.0809082	H	0.478299	-8.4601736	3.3303593
C	1.7279761	6.7326315	-3.2974512	C	-1.7279761	-4.7978473	-1.8143301
H	1.1770391	7.5149176	-3.8138562	C	-3.1220282	-4.7123053	-1.9747641
C	1.0438091	5.8283194	-2.4842981	H	-3.6738713	-3.9417573	-1.442452
H	-0.03423	5.9104014	-2.3728731	C	-3.8091323	-5.6199784	-2.7844141
C	-3.0381072	2.1694382	-0.1895009	H	-4.8885553	-5.5392644	-2.8873231
H	-3.6800163	3.0319992	-0.0444859	C	-3.1146722	-6.6332365	-3.4510172
C	-3.6234173	0.9038871	-0.402504	H	-3.6482963	-7.3403985	-4.0809082
H	-4.7061073	0.8094341	-0.429526	C	-1.7279761	-6.7326315	-3.2974512
C	-2.8293542	-0.212352	-0.496217	H	-1.1770391	-7.5149176	-3.8138562
H	-3.2913662	-1.1921801	-0.540611	C	-1.0438091	-5.8283194	-2.4842981
C	-1.4048421	-0.115126	-0.478689	H	0.03423	-5.9104014	-2.3728731

Sum of electronic and thermal Free Energies = -2075.299739

SUPPORTING INFORMATION

Table S17. Cartesian coordinates of the optimized geometry (B3LYP/6-31+G(d)) of C_i-symmetric *transoid* **1** and its Gibbs free energy.

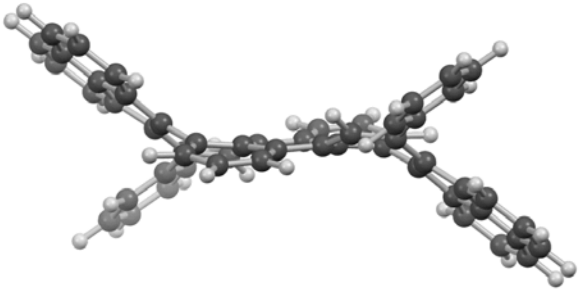

Atoms	X	Y	Z	Atoms	X	Y	Z
C	-3.7028895	-1.1545193	-0.5078273	C	1.2812434	-0.6446913	-0.125095
C	-2.3487422	-1.3407842	-1.0492279	C	1.1952611	0.6059468	0.5494691
C	-1.1952611	-0.6059468	-0.5494691	C	2.3487422	1.3407842	1.0492279
C	-1.2812434	0.6446913	0.125095	C	2.1473424	2.3865009	1.9384579
C	-2.415706	1.5555639	-0.0025244	H	3.0093697	2.8899744	2.362746
H	-2.1144011	2.6002589	-0.0221848	C	0.85216	2.8259243	2.2860294
C	-3.7503017	1.3471389	-0.1882337	H	0.727733	3.6163986	3.0217463
C	-4.3441756	0.022675	-0.1528884	C	-0.2403159	2.284053	1.6576492
C	-5.7069082	-0.3024599	0.3310861	H	-1.2317197	2.6476068	1.9032574
C	-5.825896	-1.7098562	0.2609868	C	-0.1099941	1.1844348	0.7525621
C	-4.6300487	-2.2821985	-0.2363431	C	2.415706	-1.5555639	0.0025244
C	-4.55346	-3.6652448	-0.2851644	H	2.1144011	-2.6002589	0.0221848
H	-3.6548256	-4.1805567	-0.6096717	C	3.7503017	-1.3471389	0.1882337
C	-5.6780029	-4.4364781	0.1272557	C	4.3441756	-0.022675	0.1528884
H	-5.6061343	-5.5200808	0.0774892	C	3.7028895	1.1545193	0.5078273
C	-6.8465847	-3.8571011	0.5954238	C	4.6300487	2.2821985	0.2363431
H	-7.6809797	-4.4823913	0.9050226	C	4.55346	3.6652448	0.2851644
C	-6.9436618	-2.438112	0.6905642	H	3.6548256	4.1805567	0.6096717
C	-8.0346649	-1.6755207	1.2008616	C	5.6780029	4.4364781	-0.1272557
H	-8.9372864	-2.1776158	1.5418677	H	5.6061343	5.5200808	-0.0774892
C	-7.9353912	-0.295167	1.2744257	C	6.8465847	3.8571011	-0.5954238
H	-8.7692219	0.2759579	1.6748218	H	7.6809797	4.4823913	-0.9050226
C	-6.7729907	0.4122999	0.8523436	C	6.9436618	2.438112	-0.6905642
H	-6.7484658	1.492642	0.9500115	C	5.825896	1.7098562	-0.2609868
C	-4.6274905	2.5318859	-0.4248413	C	5.7069082	0.3024599	-0.3310861
C	-4.5505545	3.6795149	0.3834128	C	6.7729907	-0.4122999	-0.8523436
H	-3.8738832	3.6882228	1.2341082	H	6.7484658	-1.492642	-0.9500115
C	-5.3509638	4.7949487	0.1251538	C	7.9353912	0.295167	-1.2744257
H	-5.2806486	5.6684222	0.7688577	H	8.7692219	-0.2759579	-1.6748218
C	-6.2447112	4.785403	-0.949337	C	8.0346649	1.6755207	-1.2008616
H	-6.8680041	5.6527675	-1.1513147	H	8.9372864	2.1776158	-1.5418677
C	-6.3336482	3.649149	-1.7601903	C	4.6274905	-2.5318859	0.4248413
H	-7.0228462	3.6320705	-2.600957	C	5.5377464	-2.5335638	1.4972753
C	-5.5377464	2.5335638	-1.4972753	H	5.61078	-1.6554779	2.1336735
H	-5.61078	1.6554779	-2.1336735	C	6.3336482	-3.649149	1.7601903
C	-2.1473424	-2.3865009	-1.9384579	H	7.0228462	-3.6320705	2.600957
H	-3.0093697	-2.8899744	-2.362746	C	6.2447112	-4.785403	0.949337
C	-0.85216	-2.8259243	-2.2860294	H	6.8680041	-5.6527675	1.1513147
H	-0.727733	-3.6163986	-3.0217463	C	5.3509638	-4.7949487	-0.1251538
C	0.2403159	-2.284053	-1.6576492	H	5.2806486	-5.6684222	-0.7688577
H	1.2317197	-2.6476068	-1.9032574	C	4.5505545	-3.6795149	-0.3834128
C	0.1099941	-1.1844348	-0.7525621	H	3.8738832	-3.6882228	-1.2341082

Sum of electronic and thermal Free Energies = -2075.295130

Table S18. Cartesian coordinates of the optimized geometry (UB3LYP/6-31+G(d)) of C_2-symmetric *cisoid* **1**$^{•+}$ and its Gibbs free energy.

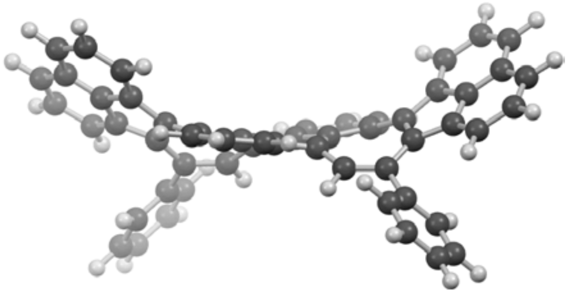

Atoms	X	Y	Z	Atoms	X	Y	Z
C	0.083077	2.875926	-0.25685	C	1.212929	-0.74324	0.244535
C	0.005421	1.456609	-0.00988	C	-0.00542	-1.45661	-0.00988
C	-1.21293	0.743236	0.244535	C	-0.08308	-2.87593	-0.25685
C	-2.35517	1.328369	0.866114	C	-1.35045	-3.48061	-0.31164
C	-2.9309	0.629294	1.466722	H	-1.41042	-4.55606	-0.42587
H	-2.84201	2.626169	0.889275	C	-2.52627	-2.73979	-0.21055
C	-2.35517	3.656925	0.021307	H	-3.48882	-3.23931	-0.27319
C	-3.14251	4.802788	-0.48556	C	-2.46426	-1.36355	-0.06597
C	-2.29996	5.491172	-1.38589	H	-3.38307	-0.78845	-0.07486
C	-1.03018	4.870605	-1.46619	C	-1.22582	-0.6967	0.053535
C	-0.11931	5.375338	-2.38898	C	2.355173	-1.32837	0.866114
C	0.851605	4.918538	-2.5523	H	2.930902	-0.62929	1.466722
H	-0.48096	6.504509	-3.16793	C	2.842011	-2.62617	0.889275
C	0.240115	6.894363	-3.88073	C	2.355173	-3.65693	0.021307
H	-1.72386	7.114652	-3.05885	C	1.068453	-3.7157	-0.55362
C	-1.96217	7.976107	-3.67772	C	1.030183	-4.8706	-1.46619
H	-2.69396	6.596989	-2.15641	C	0.11931	-5.37534	-2.38898
C	-4.0288	7.05504	-1.96752	H	-0.8516	-4.91854	-2.5523
C	-4.3918	7.916715	-2.52206	C	0.480961	-6.50451	-3.16793
H	-4.86826	6.390394	-1.08644	H	-0.24011	-6.89436	-3.88073
C	-5.88881	6.740362	-0.95972	C	1.723865	-7.11465	-3.05885
H	-4.4483	5.253044	-0.34426	H	1.962173	-7.97611	-3.67772
C	-5.15964	4.7639	0.311341	C	2.693959	-6.59699	-2.15641
H	-3.9779	2.909222	1.810466	C	2.299961	-5.49117	-1.38589
C	-5.11437	2.082681	1.860918	C	3.142514	-4.80279	-0.48556
C	-5.209	1.251804	1.166296	C	4.448301	-5.25304	-0.34426
H	-6.14415	2.343088	2.767006	H	5.159644	-4.7639	0.311341
C	-7.02324	1.70458	2.781425	C	4.868261	-6.39039	-1.08644
H	-6.04675	3.423566	3.6484	H	5.88881	-6.74036	-0.95972
C	-6.84528	3.62254	4.357725	C	4.028799	-7.05504	-1.96752
H	-4.91789	4.24875	3.61196	H	4.391799	-7.91672	-2.52206
C	-4.83226	5.086342	4.29882	C	3.977902	-2.90922	1.810466
H	-3.89675	4.000579	2.694968	C	5.11437	-2.08268	1.860918
C	-3.0219	4.644851	2.672498	H	5.208996	-1.2518	1.166296
H	1.350447	3.48061	-0.31164	C	6.144151	-2.34309	2.767006
C	1.410417	4.556065	-0.42587	H	7.023236	-1.70458	2.781425
H	2.52627	2.739788	-0.21055	C	6.046751	-3.42357	3.6484
C	3.488821	3.239311	-0.27319	H	6.845278	-3.62254	4.357725
H	2.464258	1.363551	-0.06597	C	4.917889	-4.24875	3.61196
C	3.383066	0.788451	-0.07486	H	4.832258	-5.08634	4.29882
H	1.225818	0.696704	0.053535	C	3.896745	-4.00058	2.694968
C	0.083077	2.875926	-0.25685	H	3.0219	-4.64485	2.672498

Sum of electronic and thermal Free Energies = -2075.099330

Table S19. Cartesian coordinates of the optimized geometry (UB3LYP/6-31+G(d)) of C_i-symmetric *transoid* **1**$^{•+}$ and its Gibbs free energy.

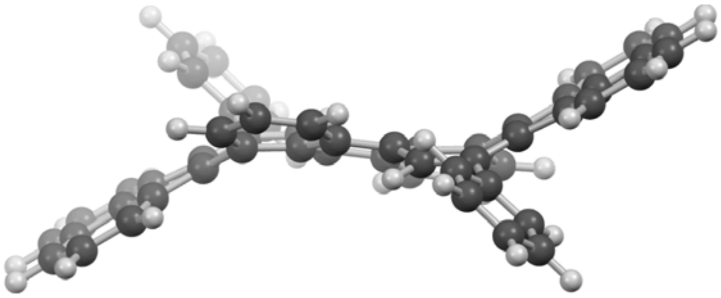

Atoms	X	Y	Z	Atoms	X	Y	Z
C	-3.7028895	-1.1545193	-0.5078273	C	1.2812434	-0.6446913	-0.125095
C	-2.3487422	-1.3407842	-1.0492279	C	1.1952611	0.6059468	0.5494691
C	-1.1952611	-0.6059468	-0.5494691	C	2.3487422	1.3407842	1.0492279
C	-1.2812434	0.6446913	0.125095	C	2.1473424	2.3865009	1.9384579
C	-2.415706	1.5555639	-0.0025244	H	3.0093697	2.8899744	2.362746
H	-2.1144011	2.6002589	-0.0221848	C	0.85216	2.8259243	2.2860294
C	-3.7503017	1.3471389	-0.1882337	H	0.727733	3.6163986	3.0217463
C	-4.3441756	0.022675	-0.1528884	C	-0.2403159	2.284053	1.6576492
C	-5.7069082	-0.3024599	0.3310861	H	-1.2317197	2.6476068	1.9032574
C	-5.825896	-1.7098562	0.2609868	C	-0.1099941	1.1844348	0.7525621
C	-4.6300487	-2.2821985	-0.2363431	C	2.415706	-1.5555639	0.0025244
C	-4.55346	-3.6652448	-0.2851644	H	2.1144011	-2.6002589	0.0221848
H	-3.6548256	-4.1805567	-0.6096717	C	3.7503017	-1.3471389	0.1882337
C	-5.6780029	-4.4364781	0.1272557	C	4.3441756	-0.022675	0.1528884
H	-5.6061343	-5.5200808	0.0774892	C	3.7028895	1.1545193	0.5078273
C	-6.8465847	-3.8571011	0.5954238	C	4.6300487	2.2821985	0.2363431
H	-7.6809797	-4.4823913	0.9050226	C	4.55346	3.6652448	0.2851644
C	-6.9436618	-2.438112	0.6905642	H	3.6548256	4.1805567	0.6096717
C	-8.0346649	-1.6755207	1.2008616	C	5.6780029	4.4364781	-0.1272557
H	-8.9372864	-2.1776158	1.5418677	H	5.6061343	5.5200808	-0.0774892
C	-7.9353912	-0.295167	1.2744257	C	6.8465847	3.8571011	-0.5954238
H	-8.7692219	0.2759579	1.6748218	H	7.6809797	4.4823913	-0.9050226
C	-6.7729907	0.4122999	0.8523436	C	6.9436618	2.438112	-0.6905642
H	-6.7484658	1.492642	0.9500115	C	5.825896	1.7098562	-0.2609868
C	-4.6274905	2.5318859	-0.4248413	C	5.7069082	0.3024599	-0.3310861
C	-4.5505545	3.6795149	0.3834128	C	6.7729907	-0.4122999	-0.8523436
H	-3.8738832	3.6882228	1.2341082	H	6.7484658	-1.492642	-0.9500115
C	-5.3509638	4.7949487	0.1251538	C	7.9353912	0.295167	-1.2744257
H	-5.2806486	5.6684222	0.7688577	H	8.7692219	-0.2759579	-1.6748218
C	-6.2447112	4.785403	-0.949337	C	8.0346649	1.6755207	-1.2008616
H	-6.8680041	5.6527675	-1.1513147	H	8.9372864	2.1776158	-1.5418677
C	-6.3336482	3.649149	-1.7601903	C	4.6274905	-2.5318859	0.4248413
H	-7.0228462	3.6320705	-2.600957	C	5.5377464	-2.5335638	1.4972753
C	-5.5377464	2.5335638	-1.4972753	H	5.61078	-1.6554779	2.1336735
H	-5.61078	1.6554779	-2.1336735	C	6.3336482	-3.649149	1.7601903
C	-2.1473424	-2.3865009	-1.9384579	H	7.0228462	-3.6320705	2.600957
H	-3.0093697	-2.8899744	-2.362746	C	6.2447112	-4.785403	0.949337
C	-0.85216	-2.8259243	-2.2860294	H	6.8680041	-5.6527675	1.1513147
H	-0.727733	-3.6163986	-3.0217463	C	5.3509638	-4.7949487	-0.1251538
C	0.2403159	-2.284053	-1.6576492	H	5.2806486	-5.6684222	-0.7688577
H	1.2317197	-2.6476068	-1.9032574	C	4.5505545	-3.6795149	-0.3834128
C	0.1099941	-1.1844348	-0.7525621	H	3.8738832	-3.6882228	-1.2341082

Sum of electronic and thermal Free Energies = -2075.295130

Table S20. Cartesian coordinates of the optimized geometry (B3LYP/6-31+G(d)) of C_2-symmetric *cisoid* $\mathbf{1}^{2+}$ and its Gibbs free energy.

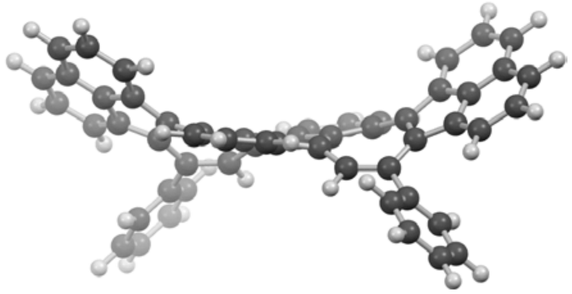

Atoms	X	Y	Z	Atoms	X	Y	Z
C	-1.297	2.29393	0.559338	C	1.296998	-2.29393	0.559338
H	-2.23357	2.002303	1.025653	H	2.233565	-2.0023	1.025653
C	-0.63559	1.264112	-0.10356	C	0.635587	-1.26411	-0.10356
C	0.776102	1.248281	-0.39638	C	-0.7761	-1.24828	-0.39638
C	1.569921	2.416412	-0.66122	C	-1.56992	-2.41641	-0.66122
C	1.057451	3.765352	-0.75126	C	-1.05745	-3.76535	-0.75126
C	-0.00087	4.347873	0.033018	C	0.000871	-4.34787	0.033018
C	-0.94802	3.644471	0.809056	C	0.948021	-3.64447	0.809056
C	1.659386	4.825578	-1.54361	C	-1.65939	-4.82558	-1.54361
C	0.99875	6.02957	-1.18939	C	-0.99875	-6.02957	-1.18939
C	0.000871	5.803633	-0.22012	C	-0.00087	-5.80363	-0.22012
C	2.59965	4.874199	-2.5807	C	-2.59965	-4.8742	-2.5807
H	3.089153	3.983072	-2.95989	H	-3.08915	-3.98307	-2.95989
C	2.888448	6.114759	-3.18466	C	-2.88845	-6.11476	-3.18466
H	3.620154	6.147384	-3.98611	H	-3.62015	-6.14738	-3.98611
C	2.250626	7.294663	-2.7997	C	-2.25063	-7.29466	-2.7997
H	2.50266	8.228544	-3.29568	H	-2.50266	-8.22854	-3.29568
C	1.254384	7.275891	-1.78963	C	-1.25438	-7.27589	-1.78963
C	0.465409	8.371782	-1.3403	C	-0.46541	-8.37178	-1.3403
H	0.627913	9.364622	-1.75104	H	-0.62791	-9.36462	-1.75104
C	-0.52326	8.164057	-0.38941	C	0.523263	-8.16406	-0.38941
H	-1.13206	9.002416	-0.06469	H	1.132055	-9.00242	-0.06469
C	-0.78376	6.885973	0.16878	C	0.783761	-6.88597	0.16878
H	-1.59191	6.78623	0.883332	H	1.591905	-6.78623	0.883332
C	1.405699	-0.03668	-0.39472	C	-1.4057	0.036682	-0.39472
C	2.771806	-0.14397	-0.6653	C	-2.77181	0.14397	-0.6653
H	3.239374	-1.12161	-0.71161	H	-3.23937	1.121609	-0.71161
C	3.551623	0.994082	-0.88863	C	-3.55162	-0.99408	-0.88863
H	4.619437	0.896653	-1.05974	H	-4.61944	-0.89665	-1.05974
C	2.961526	2.243298	-0.88069	C	-2.96153	-2.2433	-0.88069
H	3.583316	3.121369	-0.99821	H	-3.58332	-3.12137	-0.99821
C	-1.72096	4.334662	1.866363	C	1.720956	-4.33466	1.866363
C	-1.02402	5.059363	2.8557	C	3.12325	-4.22683	1.962366
H	0.058311	5.137458	2.80414	H	3.685005	-3.70348	1.192954
C	-1.71111	5.643898	3.917622	C	3.809064	-4.84224	3.008231
H	-1.16023	6.178318	4.686141	H	4.892135	-4.77666	3.055147
C	-3.10481	5.543763	3.992983	C	3.104813	-5.54376	3.992983
H	-3.6399	6.011167	4.814536	H	3.639901	-6.01117	4.814536
C	-3.80906	4.842243	3.008231	C	1.711112	-5.6439	3.917622
H	-4.89214	4.776664	3.055147	H	1.160235	-6.17832	4.686141
C	-3.12325	4.226828	1.962366	C	1.024019	-5.05936	2.8557
H	-3.68501	3.703476	1.192954	H	-0.05831	-5.13746	2.80414

Sum of electronic and thermal Free Energies = -2074.802771

SUPPORTING INFORMATION

Table S21. Cartesian coordinates of the optimized geometry (B3LYP/6-31+G(d)) of C_i-symmetric *transoid* **1**$^{2+}$ and its Gibbs free energy.

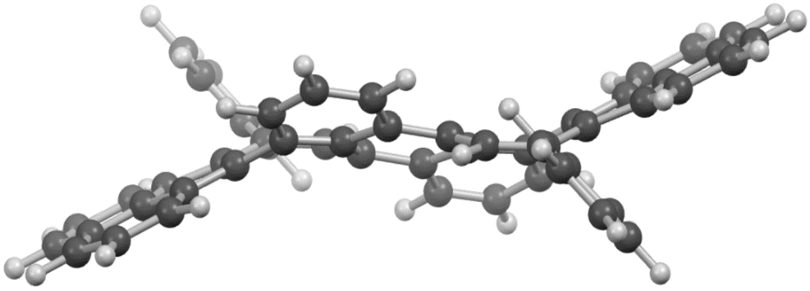

Atoms	X	Y	Z	Atoms	X	Y	Z
C	-2.3820244	1.4939161	0.1255523	C	2.3820244	-1.4939161	-0.1255523
H	-2.1190503	2.5404993	0.2325158	H	2.1190503	-2.5404993	-0.2325158
C	-1.2911652	0.6169767	0.2098727	C	1.2911652	-0.6169767	-0.2098727
C	-1.2513618	-0.684339	-0.4031554	C	1.2513618	0.684339	0.4031554
C	-2.413134	-1.4457867	-0.7887997	C	2.413134	1.4457867	0.7887997
C	-3.7538404	-1.2137399	-0.3034293	C	3.7538404	1.2137399	0.3034293
C	-4.3960716	0.0491069	-0.0747911	C	4.3960716	-0.0491069	0.0747911
C	-3.7604239	1.3095778	-0.1159988	C	3.7604239	-1.3095778	0.1159988
C	-4.7431636	-2.2617126	-0.0984466	C	4.7431636	2.2617126	0.0984466
C	-5.9617607	-1.614292	0.2277775	C	5.9617607	1.614292	-0.2277775
C	-5.8133555	-0.2124205	0.2354313	C	5.8133555	0.2124205	-0.2354313
C	-4.7083858	-3.659735	-0.0470482	C	4.7083858	3.659735	0.0470482
H	-3.7957349	-4.2222445	-0.2163171	H	3.7957349	4.2222445	0.2163171
C	-5.88946	-4.3612333	0.2735274	C	5.88946	4.3612333	-0.2735274
H	-5.8591705	-5.4459929	0.3075498	H	5.8591705	5.4459929	-0.3075498
C	-7.0859745	-3.706821	0.5674421	C	7.0859745	3.706821	-0.5674421
H	-7.9703049	-4.2886468	0.8145032	H	7.9703049	4.2886468	-0.8145032
C	-7.1461513	-2.2887626	0.5738017	C	7.1461513	2.2887626	-0.5738017
C	-8.261992	-1.4745699	0.9172713	C	8.261992	1.4745699	-0.9172713
H	-9.2117643	-1.9340906	1.1774029	H	9.2117643	1.9340906	-1.1774029
C	-8.1292971	-0.0930408	0.9347878	C	8.1292971	0.0930408	-0.9347878
H	-8.9820279	0.5180147	1.2147275	H	8.9820279	-0.5180147	-1.2147275
C	-6.910575	0.557273	0.6117008	C	6.910575	-0.557273	-0.6117008
H	-6.8638902	1.6383888	0.6708847	H	6.8638902	-1.6383888	-0.6708847
C	0.0435346	-1.2018237	-0.7442837	C	-0.0435346	1.2018237	0.7442837
C	0.1378294	-2.2573285	-1.6582162	C	-0.1378294	2.2573285	1.6582162
H	1.1080645	-2.5524891	-2.0408254	H	-1.1080645	2.5524891	2.0408254
C	-0.9987962	-2.9058518	-2.1450934	C	0.9987962	2.9058518	2.1450934
H	-0.9021689	-3.6899381	-2.8898193	H	0.9021689	3.6899381	2.8898193
C	-2.2437946	-2.5438875	-1.6687433	C	2.2437946	2.5438875	1.6687433
H	-3.1246942	-3.0567717	-2.0344713	H	3.1246942	3.0567717	2.0344713
C	-4.5555029	2.5415222	-0.3367752	C	4.5555029	-2.5415222	0.3367752
C	-5.419849	2.6102211	-1.4486533	C	5.419849	-2.6102211	1.4486533
H	-5.5214974	1.7519183	-2.1067708	H	5.5214974	-1.7519183	2.1067708
C	-6.1159948	3.7841698	-1.7297908	C	6.1159948	-3.7841698	1.7297908
H	-6.7592243	3.8322594	-2.6036618	H	6.7592243	-3.8322594	2.6036618
C	-5.9873434	4.8960485	-0.8900572	C	5.9873434	-4.8960485	0.8900572
H	-6.5409669	5.8057649	-1.1037111	H	6.5409669	-5.8057649	1.1037111
C	-5.1460838	4.8344235	0.2260163	C	5.1460838	-4.8344235	-0.2260163
H	-5.0566105	5.6899474	0.8891201	H	5.0566105	-5.6899474	-0.8891201
C	-4.4219155	3.6731359	0.4923371	C	4.4219155	-3.6731359	-0.4923371
H	-3.7888562	3.6303516	1.3749986	H	3.7888562	-3.6303516	-1.3749986

Sum of electronic and thermal Free Energies = -2074.798865

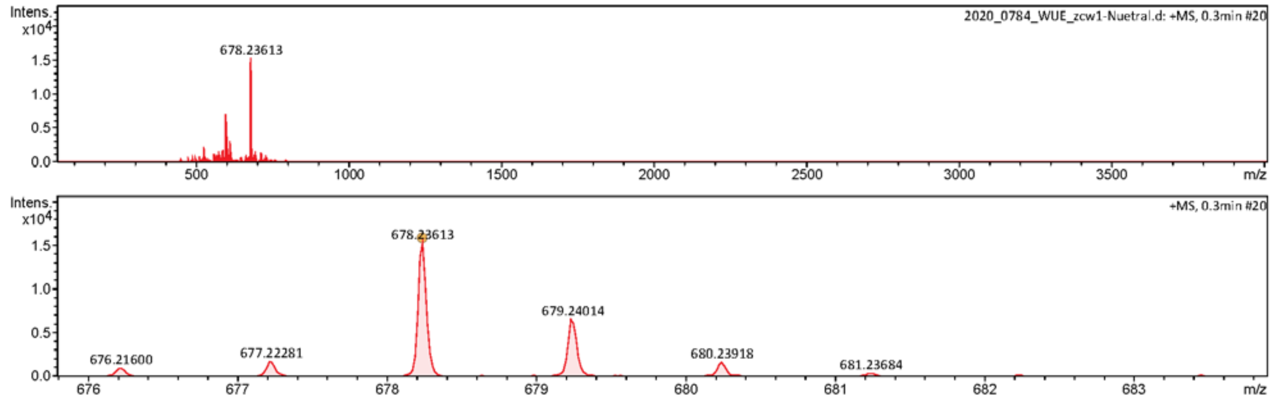

Meas. m/z	#	Ion Formula	m/z	err [ppm]	mSigma	# mSigma	Score	rdb	e⁻ Conf	N-Rule
678.23613	1	C54H30	678.23420	-2.84	85.2	1	100.00	40.0	odd	ok

Figure S16. HRMS (ESI, positive mode, in acetonitrile/chloroform) spectrum of neutral species **1**.
(Top) the whole measurement region. (Down) the expanded product peaks.

^{1}H, ^{13}C, ^{1}H-^{1}H COSY, ^{1}H-^{1}H NOESY, ^{1}H-^{13}C HSQC, and ^{1}H-^{13}C HMBC NMR Spectra

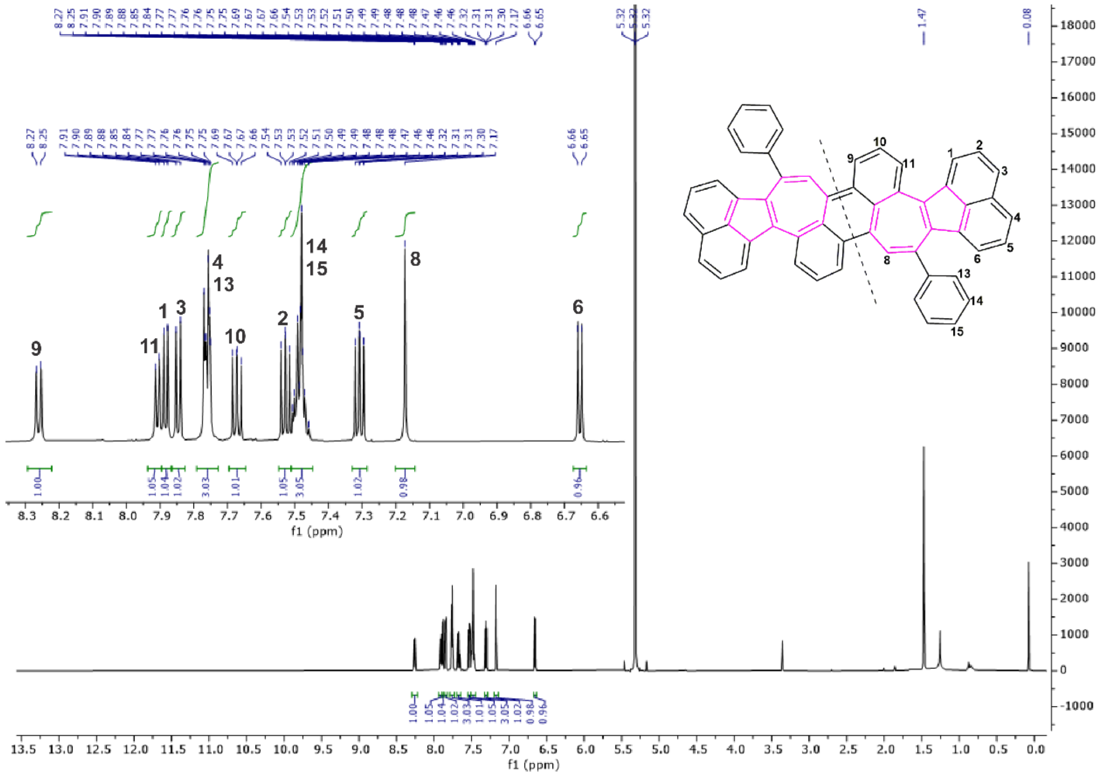

Figure S17. ^{1}H NMR spectrum of **1** in CD_2Cl_2 (600 MHz, 295 K).

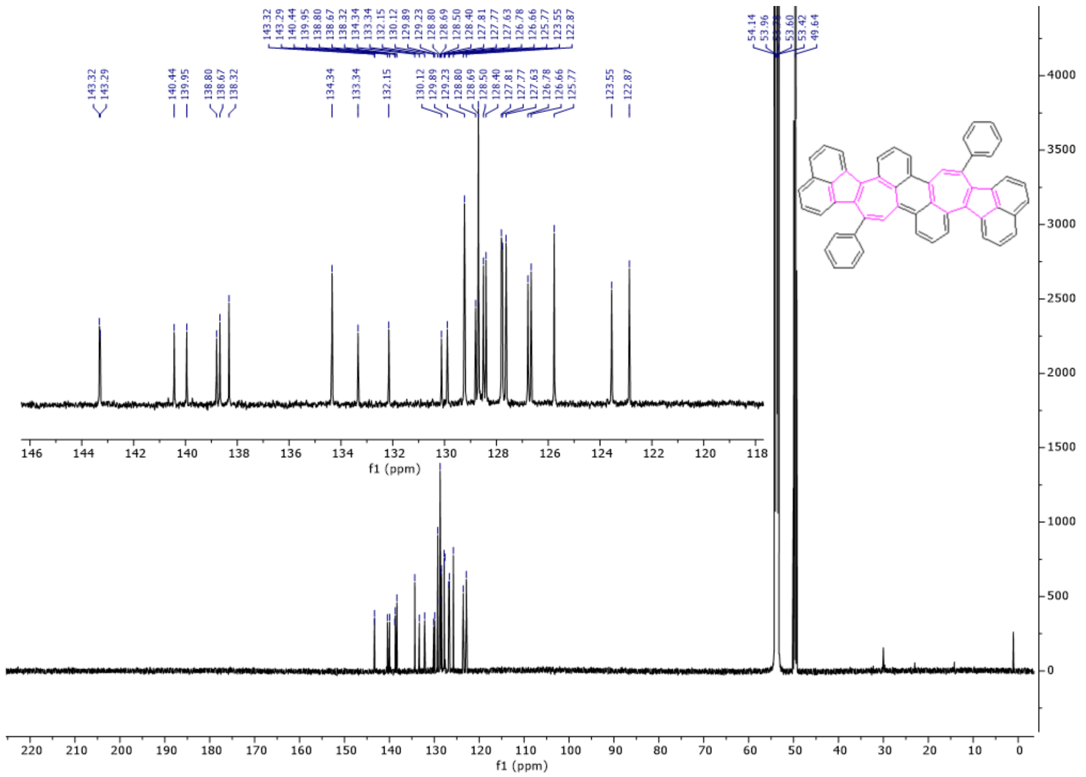

Figure S18. ^{13}C{^{1}H} NMR spectrum of **1** in CD_2Cl_2 (151 MHz, 295 K).

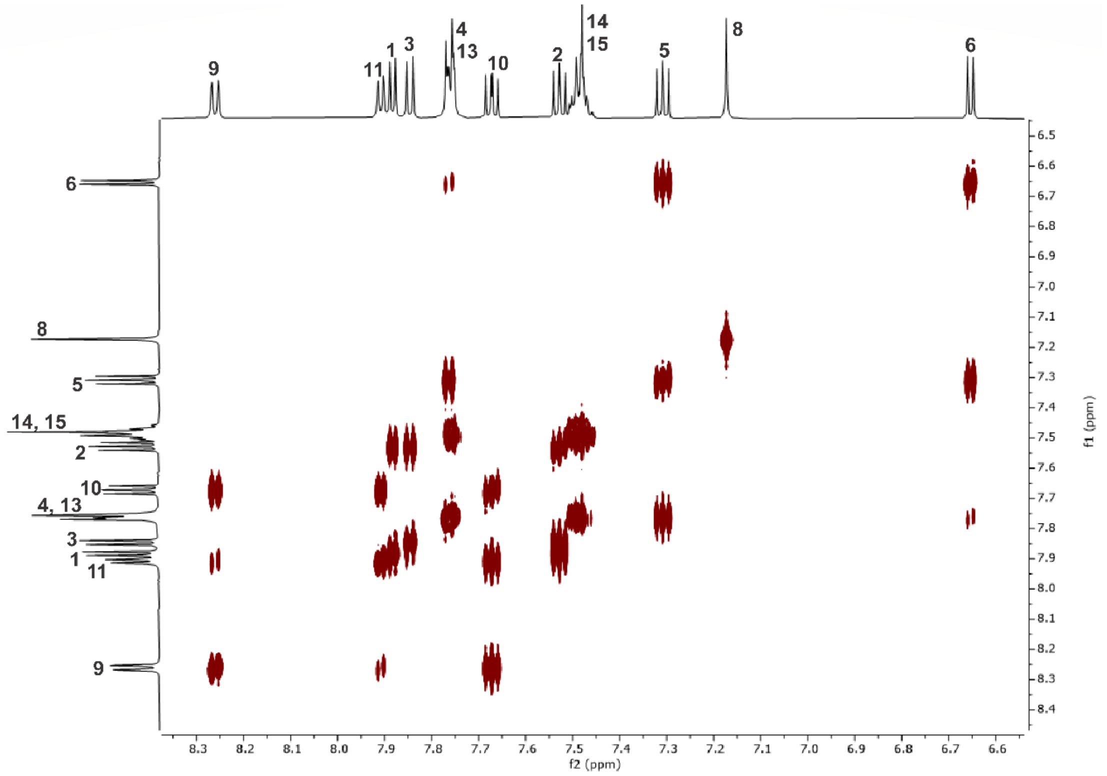

Figure S19. ^{1}H-^{1}H COSY NMR spectrum of **1** in CD_2Cl_2 (600 MHz, 295 K).

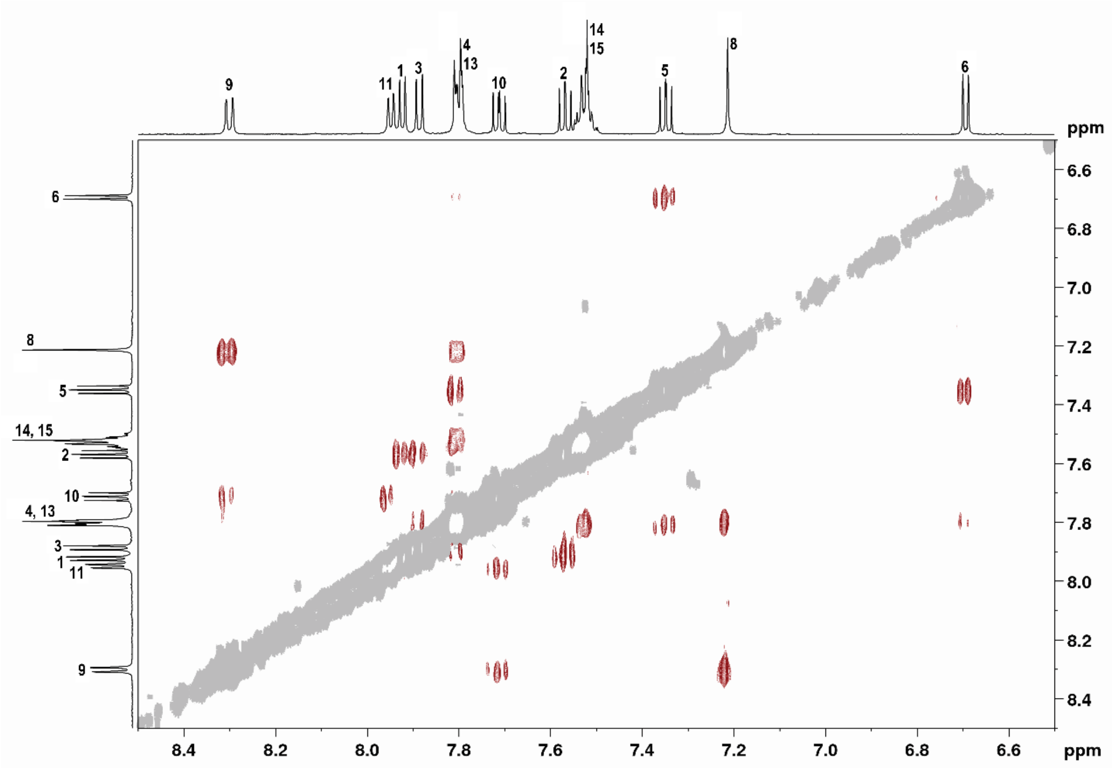

Figure S20. ^{1}H-^{1}H NOESY NMR spectrum of **1** in CD_2Cl_2 (400 MHz, 295 K).

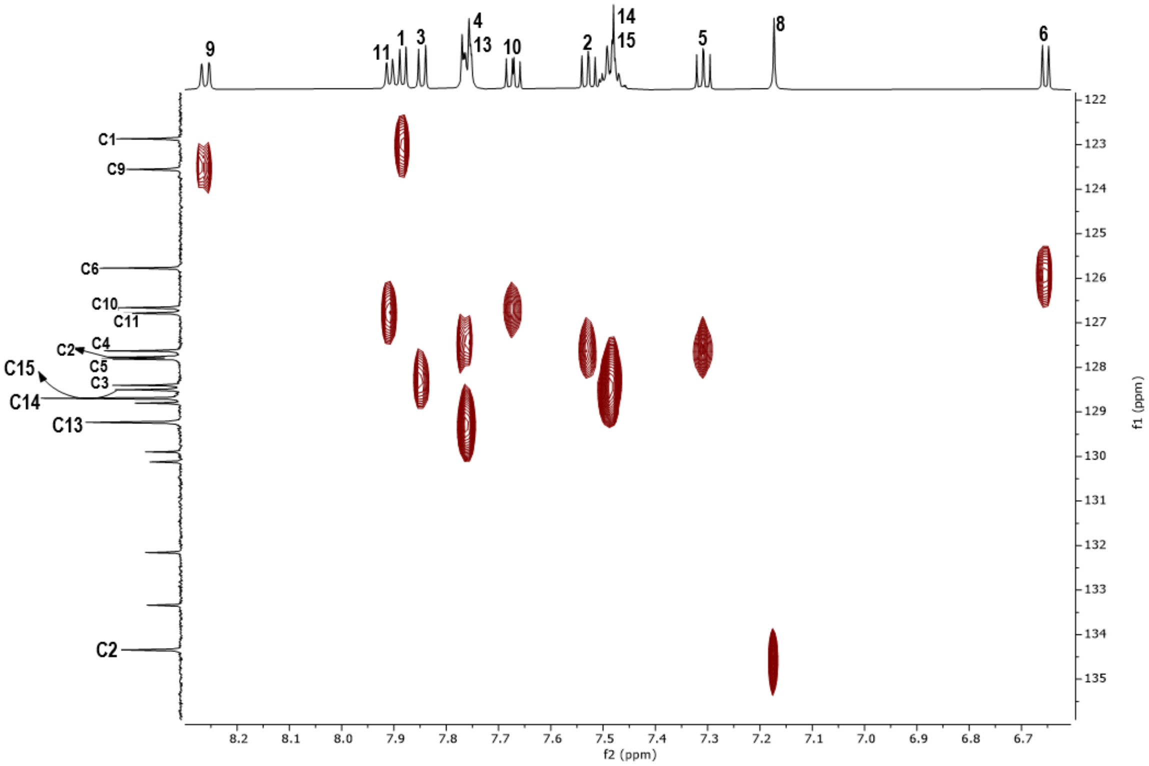

Figure S21. ^{1}H-^{13}C HSQC NMR spectrum of **1** in CD_2Cl_2 (600 MHz, 295 K).

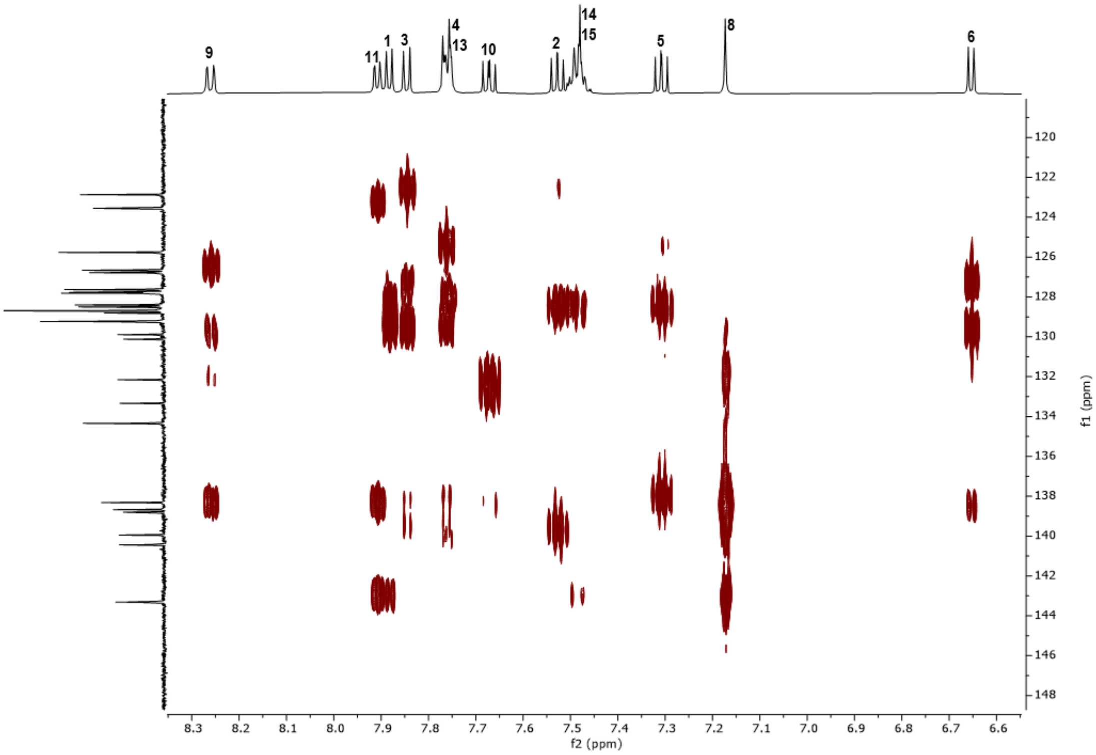

Figure S22. ^{1}H-^{13}C HMBC NMR spectrum of **1** in CD_2Cl_2 (600 MHz, 295 K).

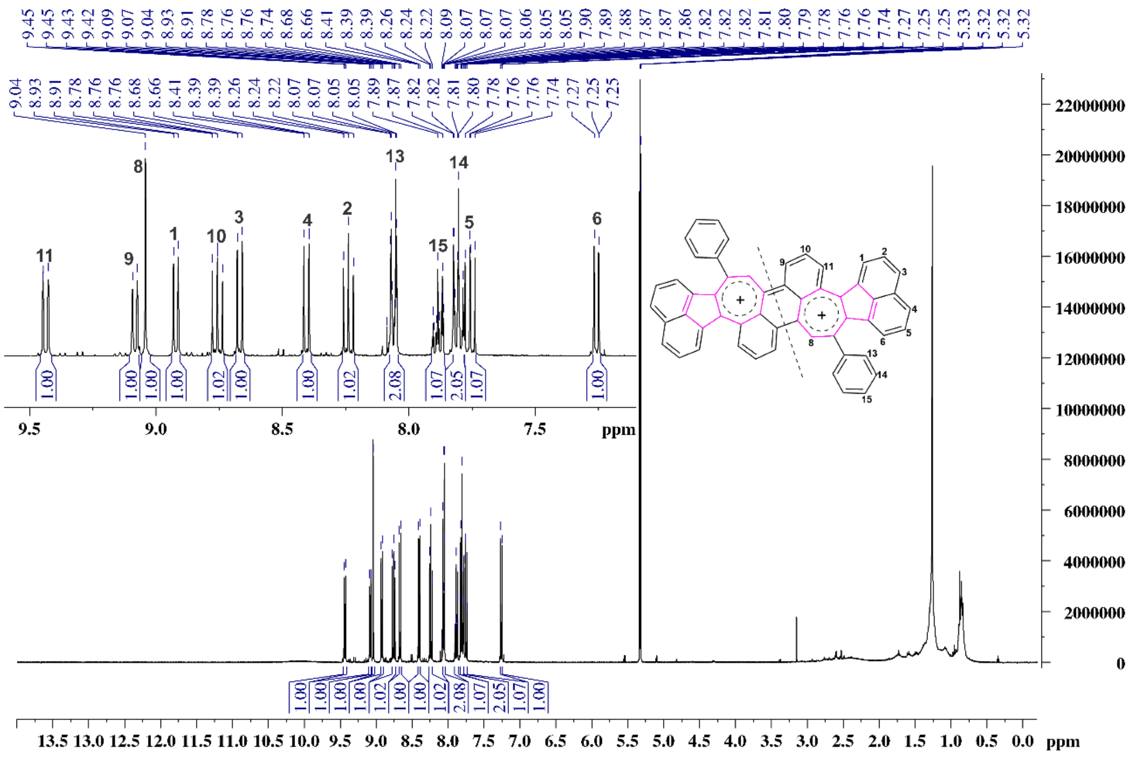

Figure S23. ^{1}H NMR spectrum of $\mathbf{1}^{2+}$ in CD_2Cl_2 (400 MHz, 295 K).

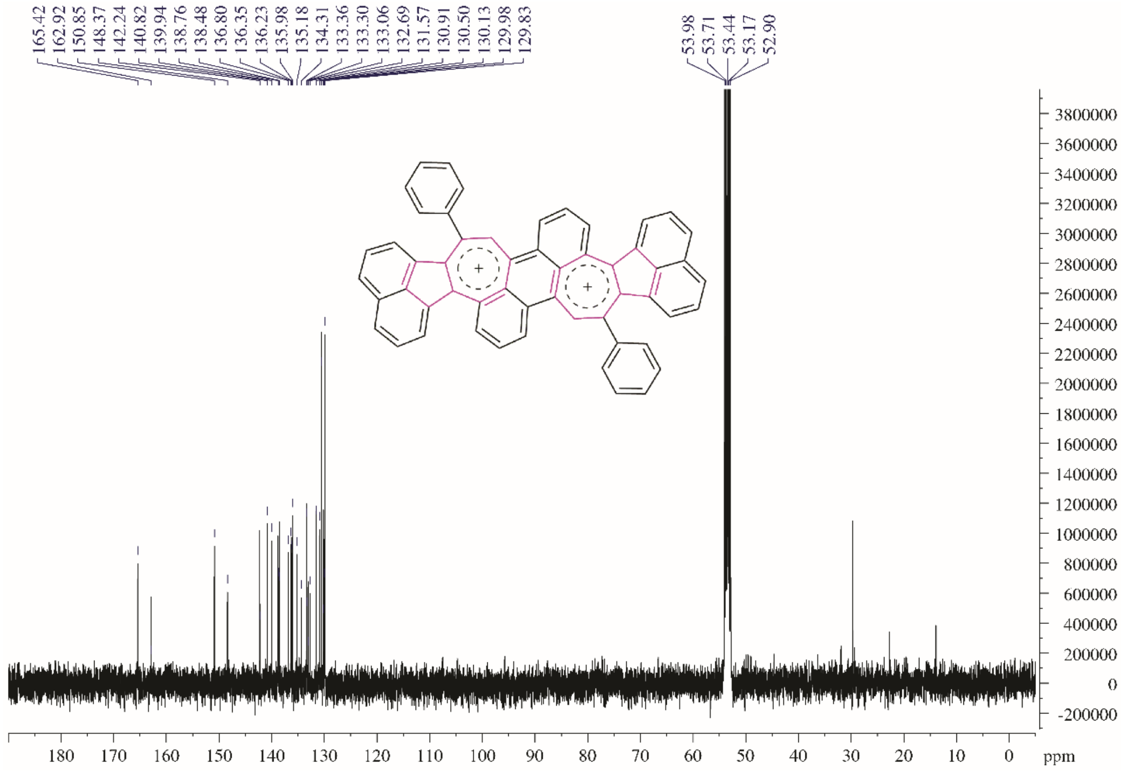

Figure S24. $^{13}C\{^{1}H\}$ NMR spectrum of $\mathbf{1}^{2+}$ in CD_2Cl_2 (101 MHz, 295 K).

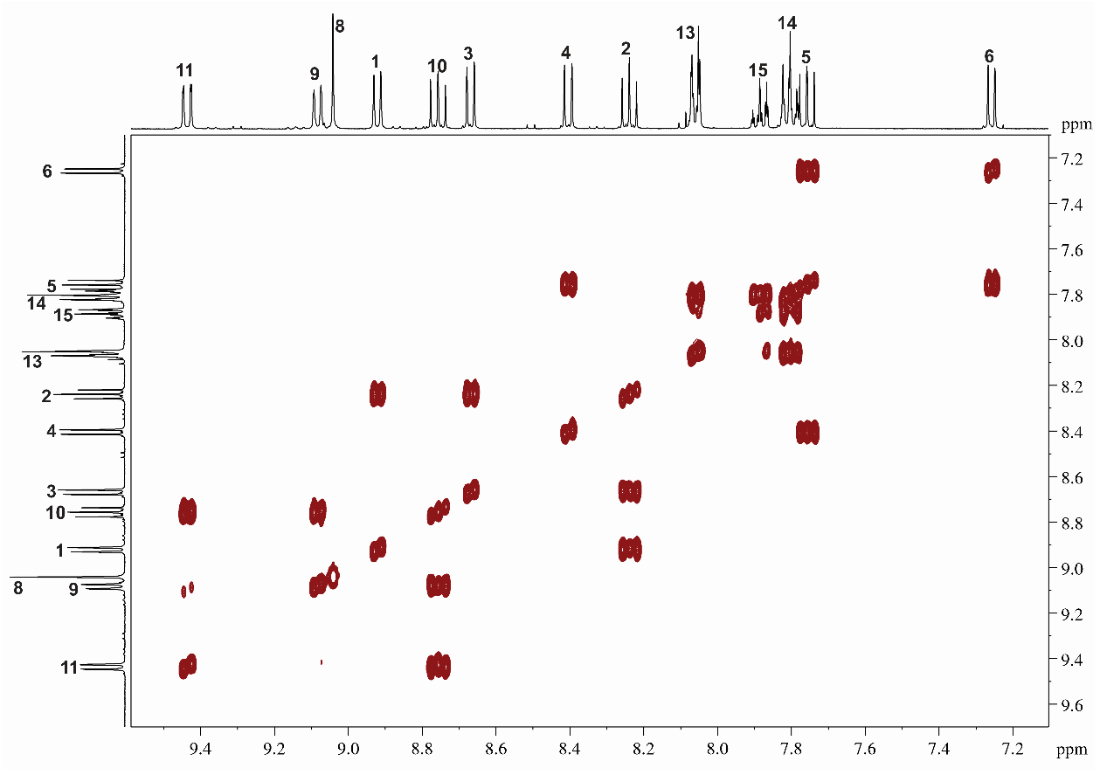

Figure S25. ^1H-^1H COSY NMR spectrum of **1**$^{2+}$ in CD_2Cl_2 (400 MHz, 295 K).

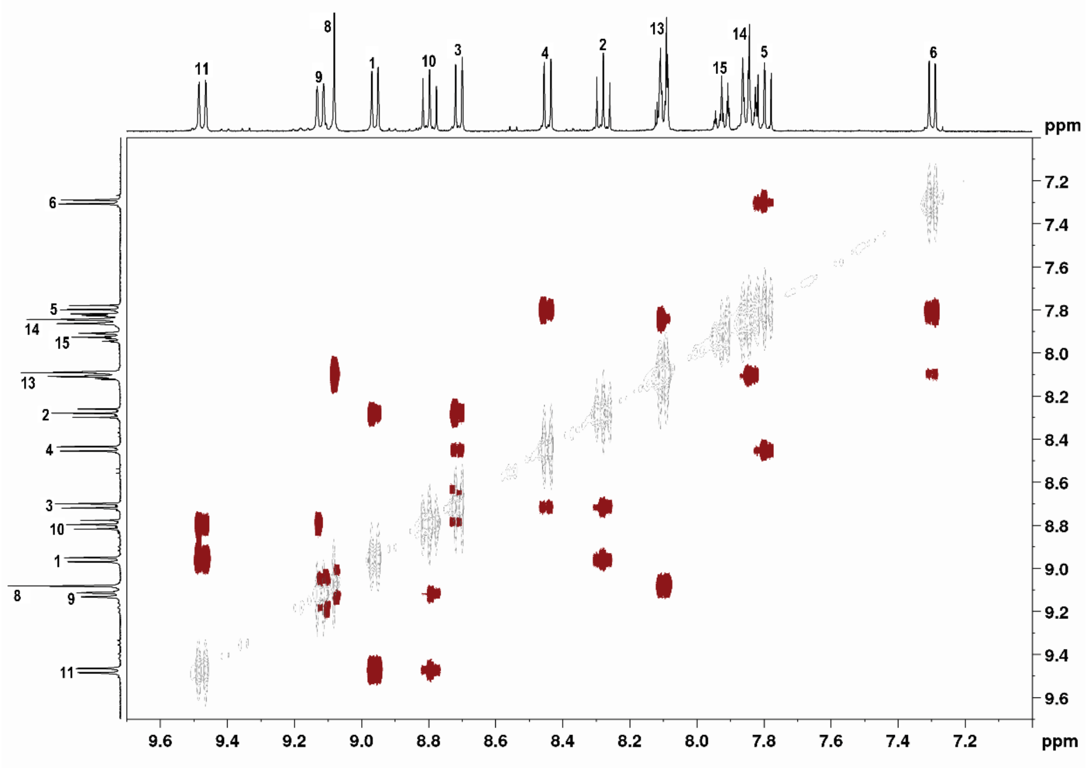

Figure S26. ^1H-^1H NOESY NMR spectrum of **1**$^{2+}$ in CD_2Cl_2 (400 MHz, 295 K).

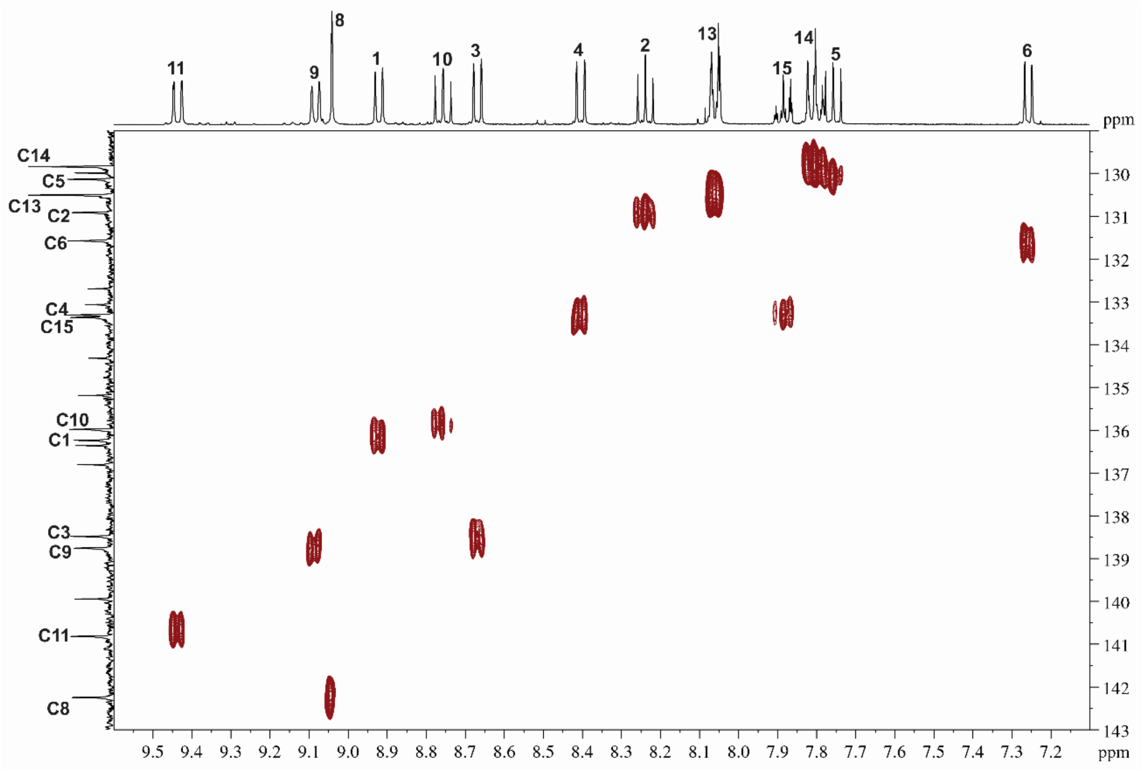

Figure S27. ^{1}H-^{13}C HSQC NMR spectrum of **1**$^{2+}$ in CD_2Cl_2 (400 MHz, 295 K).

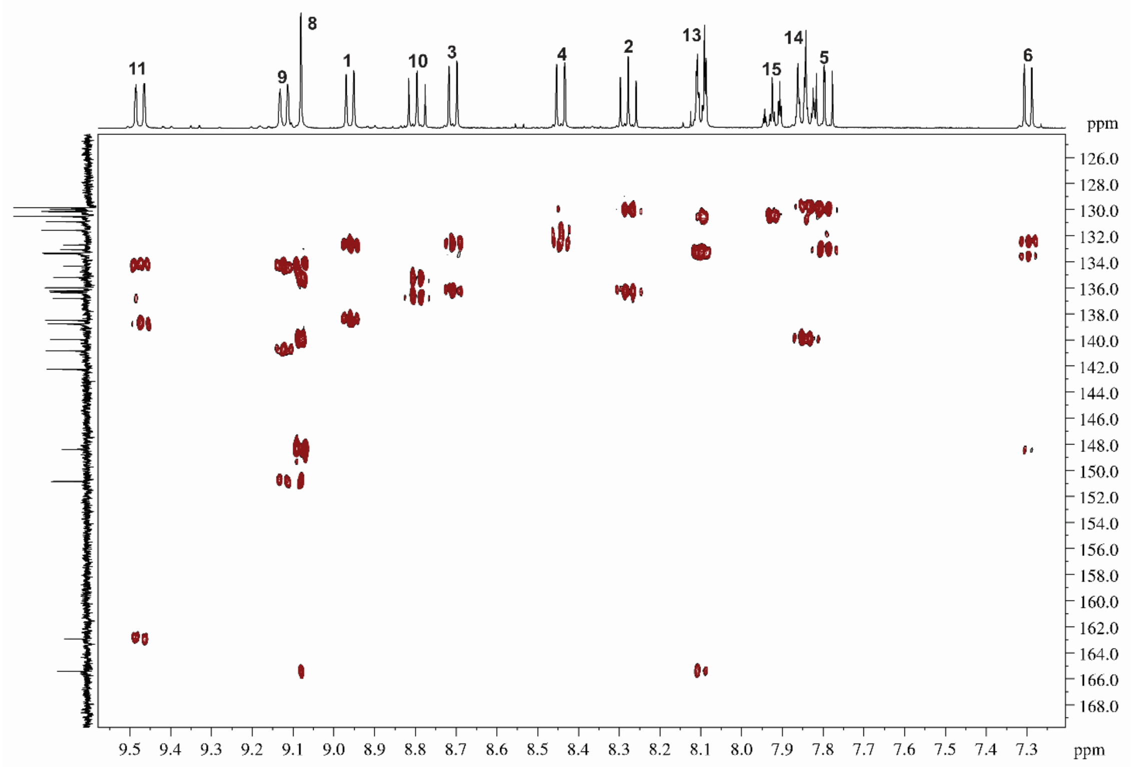

Figure S28. ^{1}H-^{13}C HMBC NMR spectrum of **1**$^{2+}$ in CD_2Cl_2 (400 MHz, 295 K).

References

[1] a) J. M. Farrell, V. Grande, D. Schmidt, F. Würthner, *Angew. Chem. Int. Ed*. **2019**, *58*, 16504-16507; b) T. Igarashi, M. Tobisu, N. Chatani, *Angew. Chem. Int. Ed*. **2017**, *56*, 2069-2073; c) E. Dimitrijević, M. Cusimano, M. S. Taylor, *Org. Biomol. Chem*. **2014**, *12*, 1391-1394.

[2] J. M. Farrell, D. Schmidt, V. Grande, F. Würthner, *Angew. Chem. Int. Ed*. **2017**, *56*, 11846-11850; *Angew. Chem*. **2017**, *129*, 12008-12012.

[3] B. M. Trost, D. R. Britelli, *J. Org. Chem*. **1967**, *32*, 2620.

[4] H. Spiesecke, W. G. Schneider, *Tetrahedron Lett*. **1961**, 14, 468–472.

[5] G. Fraenkel, R. E. Carter, A. McLachlan, and J. H. Richards, *J. Am. Chem. Soc*. **1960**, *82*, 5846–5850.

[6] a) B. Eliasson, U. Edlund, K. Müllen, *J. Chem. Soc., Perkin Trans. 2*, **1986**, 937–940; b) D. H. O'Brien, A. J. Hart, C. R. Russell, *J. Am. Chem. Soc*. **1975**; c) *97*, 44104412; U. Edlund, B. Eliasson, *J. Chem. Soc., Chem. Commun*. **1982**, 950-952; d) K. Müllen, *Chem. Rev*. **1984**, *84*, 603–646.

[7] A. L. Spek, *Acta Cryst*. C **2015**, *71*, 9–18.

[8] A. L. Spek, *J. Appl. Cryst*. **2003**, *36*, 7–13.

[10] F. H. Allen, O. Kennard, D. G. Watson, L. Brammer, A. G. Orpen, R. Taylor, *J. Chem. Soc., Perkin Trans. 2* **1987**, *0*, S1-S19.

[11] R. A. Wood, T. R. Welberry, A. D. Rae, *J. Chem. Soc. Perkin Trans. 2* **1985**, 451-456.

[12] T. Lu, F. Chen, *J. Comput. Chem.* **2012**, *33*, 580–592.

[13] R. W. Miller, A. K. Duncan, S. T. Schneebeli, D. L. Gray, A. C. Whalley, *Chem. Eur. J.* **2014**, *20,* 3705–3711.

[14] Gaussian 16, Revision B.01, M. J. Frisch, G. W. Trucks, H. B. Schlegel, G. E. Scuseria, M. A. Robb, J. R. Cheeseman, G. Scalmani, V. Barone, G. A. Petersson, H. Nakatsuji, X. Li, M. Caricato, A. V. Marenich, J. Bloino, B. G. Janesko, R. Gomperts, B. Mennucci, H. P. Hratchian, J. V. Ortiz, A. F. Izmaylov, J. L. Sonnenberg, D. Williams-Young, F. Ding, F. Lipparini, F. Egidi, J. Goings, B. Peng, A. Petrone, T. Henderson, D. Ranasinghe, V. G. Zakrzewski, J. Gao, N. Rega, G. Zheng, W. Liang, M. Hada, M. Ehara, K. Toyota, R. Fukuda, J. Hasegawa, M. Ishida, T. Nakajima, Y. Honda, O. Kitao, H. Nakai, T. Vreven, K. Throssell, J. A. Montgomery, Jr., J. E. Peralta, F. Ogliaro, M. J. Bearpark, J. J. Heyd, E. N. Brothers, K. N. Kudin, V. N. Staroverov, T. A. Keith, R. Kobayashi, J. Normand, K. Raghavachari, A. P. Rendell, J. C. Burant, S. S. Iyengar, J. Tomasi, M. Cossi, J. M. Millam, M. Klene, C. Adamo, R. Cammi, J. W. Ochterski, R. L. Martin, K. Morokuma, O. Farkas, J. B. Foresman, and D. J. Fox, Gaussian, Inc., Wallingford CT, 2016.

[15] Z. Chen, C. S. Wannere, C. Corminboeuf, R. Puchta, P. V. R. Schleyer, *Chem. Rev.* **2005**, *105*, 3842–3888.
